# New insights into archaeological textiles (1000–1450AD) from the coastal region of the Atacama Desert: Preliminary evidence of a cochineal and shellfish purple dye combination

**DOI:** 10.1371/journal.pone.0325623

**Published:** 2025-06-04

**Authors:** José J. Cárcamo-Vega, Marcela Sepúlveda, Edgar Casanova-González, Sebastián Gutiérrez, Alejandro Mitrani, Elard J. Dauelsberg, Álvaro E. Aliaga, Cecilia Lemp, José Luis Ruvalcaba-Sil

**Affiliations:** 1 Universidad de Tarapacá, Arica, Chile; 2 Departamento de Ciencias Sociales, Facultad de Ciencias Sociales, Universidad de Tarapacá, Iquique, Chile; 3 CONAHCyT—Laboratorio Nacional de Ciencias para la Investigación y Conservación del Patrimonio Cultural, Instituto de Física, Universidad Nacional Autónoma de México, Mexico City, Mexico; 4 Laboratorio de Análisis e Investigaciones Arqueométricas, Departamento de Antropología, Universidad de Tarapacá, Arica, Chile; 5 Laboratorio Nacional de Ciencias para la Investigación y Conservación del Patrimonio Cultural, Instituto de Física, Universidad Nacional Autónoma de México, Mexico City, Mexico; 6 Laboratorio de Espectroscopía Vibracional, Departamento de Química, Facultad de Ciencias, Universidad de Chile, Ñuñoa, Chile; 7 Independent researcher, Santiago, Chile; Universidad de Sevilla, SPAIN

## Abstract

A multi-instrumental and non-destructive approach was used to integrally analyze four archaeological textiles from the Pre-Columbian Playa Miller-3 funerary site (1100–1450 AD) located on the coast of the Atacama Desert. The protocol included a fiber washing process with a dilute Triton X100 surfactant to remove the excess metallic components and impurities adhered to the surface of the dyed fibers. The use of animal fibers was confirmed via optical microscopy, Scanning Electron Microscopy (SEM) and Fourier Transform Infrared Spectroscopy (FTIR). Using X-ray fluorescence (XRF) and comparing the elemental profiles of washed and unwashed textile fibers with those of soils from the archaeological site, we obtained elemental information related to potential mordants used in the dyeing process and detected the presence of bromine in some textile samples. Surface-Enhanced Raman Spectroscopy (SERS) using gold nanostructures identified the use of carminic acid and suggested a dye mixture composed of cochineal (carminic acid) and shellfish purple (dibromoindigo), which has not been previously reported for the Atacama Desert. This work provides new insights into pre-Columbian ancestral knowledge involved in the textile technology of the coastal population from this southern Andean region.

## 1. Introduction

In the Atacama Desert of northern Chile, the inhabitants of the coast and low valleys have a millennial textile tradition dating back to the Archaic period, between 10500 and 3500 BP, during which they produced textiles with plant fibers from *Typha* sp. and *Schinoeplectus aff.* *S* *americanus* [1–3]. Gradually, textile technology incorporated other raw materials (cotton and camelid wool) [4] with new weaving techniques, such as the loom and the dyeing of fibers [5]. Studies on the archaeological textiles concerned techniques, shapes, styles, and the iconography of pieces from huge cemeteries associated with different moments of the pre-Columbian sequence of northern Chile [6–8]. In Chile, few works have focused on textile technology, including the chemical characterization of materials, such as fibers, dyes, and mordants [5,9–12]. These ancient technologies involve complex immaterial, mathematical knowledge and weaving skills. Decisions consist of knowing how to choose the size, shape, designs, iconography, and colors, among many other things, as well as mastering the properties associated with the raw materials used to dye the fibers and to make the textiles [13].

In the last three decades, interdisciplinary efforts in the Andes region of South America have addressed the study of colored textile materials in greater depth. However, they are still generally focused on identifying dyes [5]. Other materials and compounds (such as fibers and mordants) are rarely analyzed [14–20], and most of the published works study the Paracas textile tradition from the southern coast of Peru [5]. Finally, a multi-instrumental approach is scarcely applied, so our comprehension of textile technologies in pre-Columbian times is still limited.

Initial conditions of light, temperature, humidity, and elemental and molecular composition used in textile production evolve into a stable thermodynamic state with the stabilization of various inorganic and organic molecular systems, which may persist for thousands of years [21–28]. The materials used and the intentionality in the fixation of color on fiber are the product of an ancient and complex textile technology that has developed over time. In particular, fixing an organic dye on an animal fiber should consider the interaction time of the material components, exposure of the yarn to specific mordants, temperature, and dispersion medium of the process, among others [13]. When studying the materiality of archaeological textiles, it is necessary to take into account that the physical-chemical characteristics of the soil and environment of the archaeological site determine the taphonomic conditions affecting materials after they were buried. In effect, we cannot discard the fact that textiles from archaeological cemeteries interact with soils and body fluids, which can alter the different textile materials. Many taphonomic factors (minerals from the soil, eventual pollution, storage conditions, among others) alter the various materials and textile preservation. We must also consider the biography of the textiles after the archaeological excavation, the manipulation in the museum, and their preservation conditions, which could have consequences on the tonalities observed [26]. However, due to the hyperarid conditions in the Atacama Desert, various molecular systems are well-preserved in archaeological materials, allowing their study with different analytical tools [5,11,21–25,27,29].

**Fig 1:**
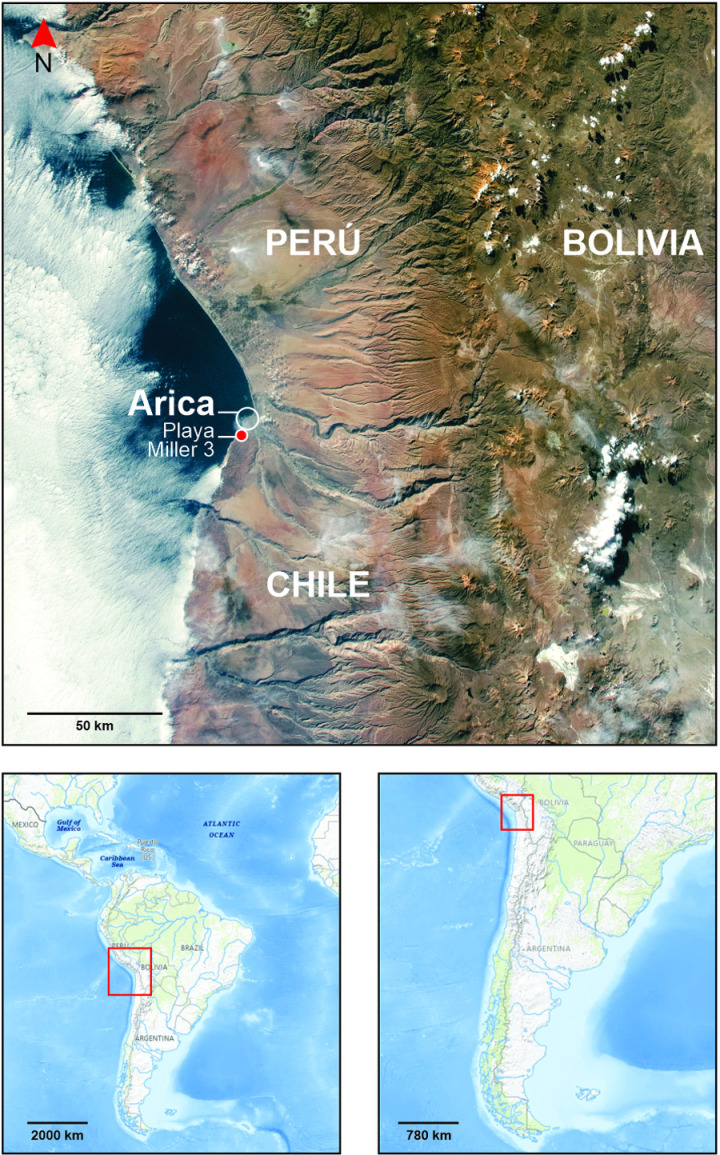
Map of the archaeological site of Playa Miller 3 near the modern city of Arica, on the coast of the Atacama Desert. Modified by the authors from NASA Earth Observatory (https://earthobservatory.nasa.gov/) and USGS National Map Viewer (https://www.usgs.gov/tools/national-map-viewer) with Corel Draw v.24.2.1.446.

In this work, we analyzed red fibers from four archaeological textiles of the Late Intermediate period (1100–1450 AD) collected from the northern coast of the Atacama Desert (Fig 1). We employed a multi-instrumental analytical methodology that combines optical microscopy (OM), Scanning Electron Microscopy (SEM), X-ray fluorescence (XRF), portable X-ray fluorescence (p-XRF), Fourier-Transform Infrared spectroscopy (FTIR), and Surface-Enhanced Raman Scattering spectroscopy (SERS) with concentrated colloidal gold nanoparticles (AuNPs). Portable XRF contributed to identifying chemical elements in the soil from the archaeological site. OM, SEM and FTIR were used to provide information on the nature of the fibers. SERS technique was also employed for identifying the dye molecules used on the yarns, an application where it has demonstrated some success on archaeological and heritage samples [5,11,30–34]. While SERS analyses can be very precise under controlled conditions, when analyzing complex materials, such as archaeological objects, it is often difficult to acquire clear and unambiguous spectra.

Before performing the SERS analyses, the textile samples were washed with a non-ionic surfactant to reduce the contents of potential contaminants present in the soil at the archaeological site and to improve the analytical protocol. In order to support the findings from SERS analyses on the archaeological samples, both undyed wool and a modern dyed cotton textile were also studied.

Finally, whenever possible, HPLC should be part of the analytical protocol for studying dyed fibers, if sampling is allowed. HPLC should provide a solid confirmation of the spectroscopic results. Unfortunately for this study, it was not possible to perform HPLC analyses at the time the archaeological fibers were available for analysis.

### 1.1. Archaeological context

Samples came from textiles excavated at Playa Miller-3 (PLM-3) archaeological site on a hillside south of the modern city of Arica (Fig 1). The first references to this site appear at the end of the 19th century [29]. Excavated by Skottsberg in the 1920s [35], it was again studied in the 1940s by Junius Bird [36] and Grete Mostny [37]. Finally, systematic excavations were carried out in the 1960s when a new road was constructed along the coast [29]. The site is the most extensive funerary context excavated on the northern Chilean coast, with 233 tombs. Today, we can still observe archaeological materials on the surface as the product of constant looting.

The entire context indicates a hunter-gatherer and fishermen population. The funerary offerings include a wide array of artifacts associated with these activities, such as “cotton canvas for fishing, wooden harpoons with lithic and bone tips, plant fiber harpoon holders, sea lion bone chippers, cactus hooks, and copper, decorated baskets of woven vegetable fiber and wooden structure, miniatures of three-masted rafts, double-bladed oars, lithic weights, bags in mesh technique” [6,7,29,38]. Additionally, pelican feathered headdresses (diadem) and the iconography displayed on specific textile garments from PLM-3, reinforce the idea of a group whose life was closely linked to the sea [38]. The funerary offerings include wood, ceramic, and gourd artifacts. They also crafted textiles with wool balls, cactus needles, and bone instruments [38]. From the 3450 objects that have been excavated at PLM-3, 33% correspond to textiles and 22% *inkuñas* [7].

The Aymara concept of “*inkuña*” designates those ritual woven sheets of rectangular shape used in coca or other ceremonies of substances like *k’oa* and *sorona* [29,39]. They were also used to transport goods of ritual and personal value, a function extended to Andean festivities until today [40,41]. The archaeological *inkuñas* were made with looms, resulting in sheets of medium dimensions (almost 60 by 60 cm). Their size made them easily transported and placed around the main funerary bundle, between garments, and over shrouds covering the dead. *Inkuñas* reached their most complex form of craft and frequency in contexts during the Late Intermediate Period, between 1100–1450 AD on the northern coast of Chile [6,7,29].

**Fig 2:**
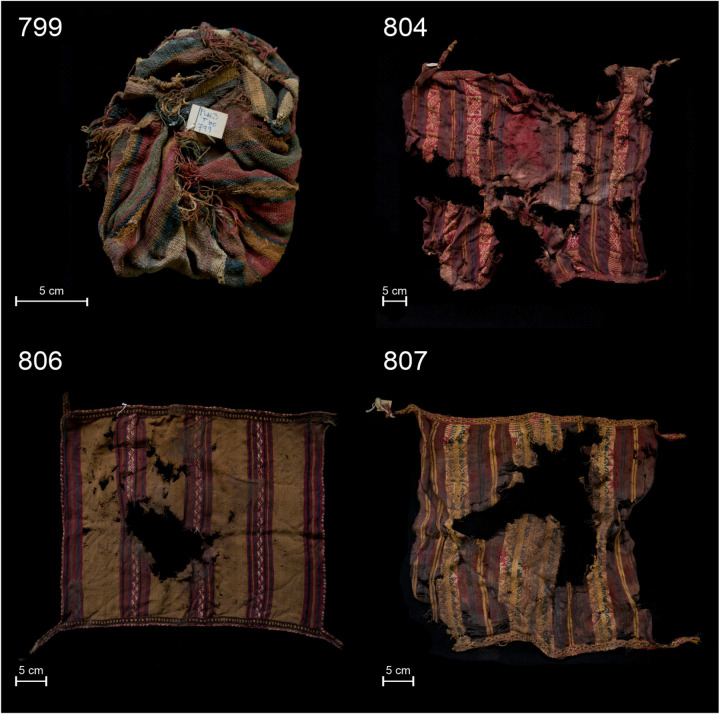
Archaeological textiles analyzed: A) 799; B) 804; C) 806; and D) 807.

A common characteristic of *inkuñas* technology is their structure in warp-faced weaves (Fig 2). These can be categorized into two groups based on color and iconography [6]. The first group features stripes and lines crafted with discontinuous and alternated warps. The second group presents stripes or bands containing varied iconography, with a predominance of complementary warps and cases of floating or non-structural and supplementary warps [6,42]. *Inkuñas* exhibit crafted edges at both ends of the warps: one of these edges is twined, which extends in “twined handles” at each of their corners [6]. These twined corners not only serve as ornamentation and as reinforcement for the sheet’s length, but also function as a strapping system, allowing the *inkuña* to easily contain different substances and small artifacts.

Another distinctive feature in the production of *inkuñas* is their diversity in designs and chromatic effects. To achieve this, weavers used rich color variations in the dyeing process. In contrast to the limited colors used in earlier periods, the yarns for *inkuñas* were dyed with blue, green, yellow, and various shades of red – ranging from red to purple – [8,42]. There is evidence of natural color yarns such as brown, ochre, white, and off-white. The use of such a broad spectrum of colors represents one of the most distinctive features of these objects, marking a qualitative leap in terms of the diversity in the design of these textiles.

In particular, burial 60 at Playa Miller 3 comprises 23 objects: raft miniatures made of three logs in an unfinished state, fishing and fish-collecting tools, ceramics, wood artifacts intended for different purposes, a wool ball, and nine *inkuñas*. Even though burial 60 does not constitute an elite funerary context, it is one of the tombs that concentrates the most significant number of *inkuñas* [6].

Four red samples were selected for analysis from the *inkuñas* discovered at burial 60: textiles 799, 804, 806, and 807 (Fig 2). The chosen *inkuñas* share common attributes regarding technique, form, and material composition: they were woven as a single cloth or sheet and used warp-faced weaving with different variations on every piece. This technique defines the verticality of the design, resulting in finished edges of twined warps, where their remaining yarns are extended as handles, with the exception of object 799, which is tied and unfinished (Fig 2A).

## 2. Samples and methods

### 2.1. Samples

**Fig 3:**
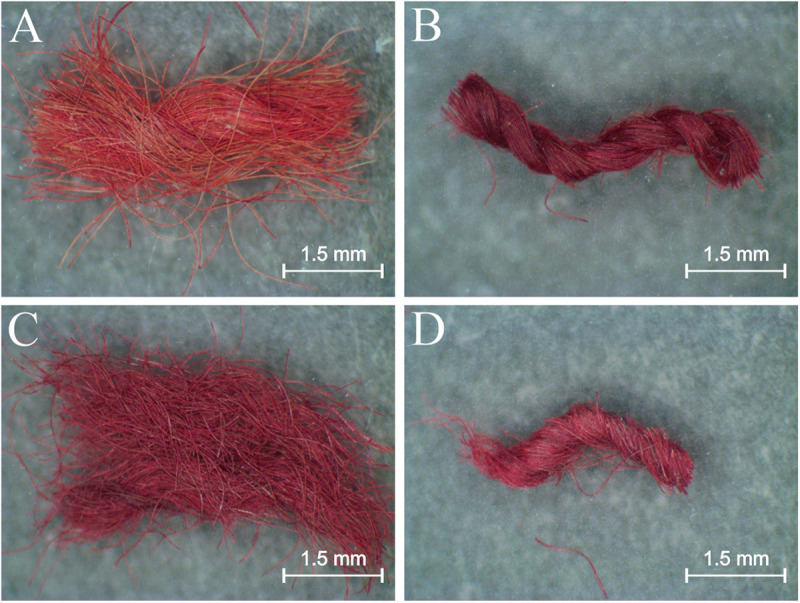
Detached samples collected from textiles 799 (A), 804 (B), 806 (C) and 807 (D).

Sampling was carried out in the textile collection depots of the Archaeological Museum of San Miguel de Azapa, University of Tarapacá, with the authorization of the National Monuments Council of Chile, notification 246/2013. During this process, the pieces were unfolded, photographed and stored in the new depots. In order to avoid intervening these unique archaeological pieces, only fragments that had come loose during the handling of the *inkuñas* were collected. Most of these detached textile threads did not present defined colors. For this study, only threads with well-defined red tones were selected, which had lengths ranging from 2.5 to 5 mm (Fig 3).

Two millimeter portions from the samples were washed according to the procedure described below. The washing procedure was carried out on 3 independent sample portions.

Additionally, a modern cotton textile dyed with shellfish purple, part of the reference materials collection of LANCIC, was available for analysis. This textile was a personal gift from Gabina Aurora Pérez Jiménez, a former researcher on Mixtec Language and Culture from the Faculty of Archaeology of Leiden University, Netherlands. Ms. Pérez Jiménez was born and raised in Chalcatongo, Oaxaca. The state of Oaxaca, Mexico, is well known for its strong dyeing tradition, and dyeing with shellfish purple is still carried out in the state’s Pacific coast.

### 2.2. Multi-instrumental protocol

The techniques applied in the present study were selected for the possibility of analyzing small samples, and for their capacity to identify fibers, mordants, and dye molecules through the combination of multiple methods. FTIR and XRF analyses on 2 mm sample portions were performed at the National Laboratory of Sciences for the Research and Conservation of Cultural Heritage (LANCIC) in Mexico. At the same time, p-XRF measurements were carried out in situ on soils from PLM-3, while Scanning Electron Microscopy (SEM) and SERS spectroscopy were performed at the Laboratory of Archaeometric Analysis and Investigation (LAIA), in Arica, Chile. Microscopic observations of the textile samples were carried out in the Vibrational Spectroscopy Laboratory, Faculty of Sciences, University of Chile, in Santiago, Chile. Additionally, Raman, SERS, XRF and Fiber Optics Reflectance Spectroscopy (FORS) analyses of wool and a dyed modern textile were also performed at LANCIC, Mexico.

At the time the archaeological fibers were analyzed, HPLC was not readily available. As will be seen below, our results indicate that HPLC would have been an important step in order to complete the analysis and should be included in future research on this subject, whenever sampling is allowed.

#### 2.2.1. Sample washing.

First, a washing protocol was applied to the archaeological textile samples. This protocol accounted for various potential contaminants present in the soil at the funerary site, which can interfere with the spectroscopic analyses. The samples were washed with a diluted non-ionic detergent (Triton™ X-100) commonly employed in cell washing [19,43,44]. Detergent solutions at low concentrations (0.01% v/v) were prepared to wash textile fragments approximately 2 mm in length inside a glass vial, which were then vortexed for 5 minutes. Subsequently, successive rinses with type 1 water (with a resistivity of 18.2 MΩ.cm at 25 °C and a total organic carbon (TOC) ≤ 5 ppb) were performed to remove any trace of impurities and detergent. The effectiveness of this washing protocol was verified through SEM examination.

#### 2.2.2. Optical microscopy.

Optical microscopic (OM) observations were performed with a ZM-4T stereo trinocular microscope (AmScope), using reflected light and 15X magnification, and a BA310 microscope (Motic), using transmitted light and 40X magnification. This allowed us to obtain and record detailed morphological information on the archaeological textile fibers, evaluate color tone, and identify particles adhered to the fibers from the original archaeological context.

For stereomicroscopic recordings under reflected light, the samples were placed on a slide and covered with a coverslip to prevent movement of the sample. For microscope observations under transmitted light, the samples were immobilized with a mounting medium (EUKITT™) on a slide and protected with a coverslip.

#### 2.2.3. Scanning Electron Microscopy.

SEM analyses were performed with a JCM-6000 tabletop microscope (JEOL). Images were acquired at high vacuum conditions, with backscattered electrons, using a 1300X magnification, with 10 and 15 kV voltage. Samples were not coated.

#### 2.2.4. Fourier-Transform Infrared spectroscopy (FTIR).

A Bruker Alpha spectrometer recorded FTIR spectra in ATR acquisition mode. The spectrometer has a CenerGlow infrared source, a diode laser, a corner-cube interferometer, and a controlled temperature DLaTGS detector. The ATR module comprises a diamond ATR crystal with a 45° incidence angle. Thirty-two scans were averaged per acquisition, with a 4 cm^−1^ resolution and a 4000–400 cm^−1^ spectral range.

#### 2.2.5. X-ray Fluorescence analysis (XRF).

Elemental analyses of the archaeological textile fibers via XRF were conducted with SANDRA, a home-built portable system [45]. This system is equipped with a Mo X-ray tube that irradiates a 1 mm diameter spot, and an Amptek X-123 SDD X-ray detector. Analytical conditions were set at 40 kV and 30 mA with a 90 s acquisition time. Spectral deconvolution was performed with PyMCA [46] to obtain elemental peak intensities. A difficulty encountered in these analyses was identifying whether the elements detected were intrinsic to the textile samples or were post-depositional contaminants, primarily soil attached to the fibers’ surface. Analyses were performed on the unwashed and washed samples to help understand the elements’ origin. This approach allowed us to observe compositional changes, where the components lost by the chemical and mechanical action of the detergent and washing process are generally related to post-depositional contaminants. Special care was taken when comparing these analyses, as the small textile samples make it impossible to ensure the same amount of sample in both studies. For this reason, X-ray intensities were normalized to the total X-ray intensity counts of each spectrum in order to take into account the mass differences among the samples and only general trends in the elemental compositions were considered.

*In situ* elemental analyses were performed on the archaeological soil at PLM-3 (S1 Fig) to gain further insight into the composition of the post-depositional contaminants. A handheld Tracer III SD XRF spectrometer from Bruker was used for *in situ* analysis, operated at 40 kV, 37.8 µA, with a 120 s acquisition time. Twenty-one measurements were taken along 2 diagonal transects over 120 m long, which allowed the full length of the excavated area at PLM-3 to be covered (S1b Fig).

#### 2.2.6. Surface-Enhanced Raman Scattering spectroscopy (SERS).

Colloidal gold nanoparticles were prepared for SERS measurements using the Turkevic method described by Aroca [47,48], by chemical reduction of HAuCl_4_ with sodium citrate. For this, 0.1 ml of HAuCl_4_ solution (4%, w/v) was added to 40 ml of triple distilled water. Subsequently, 1 ml of trisodium citrate solution (1%, w/v) was added dropwise under constant stirring. Finally, the resulting mixture was boiled for 5 min. The colloidal gold nanoparticles show an extinction maximum centered at 527 nm, which is characteristic of these nanostructured systems (S2 Fig).

The synthesis of colloidal silver nanostructures was carried out using the method described by Leopold N. and Lendl B [49]. In particular, the colloidal metallic silver was obtained by reducing 10 ml of silver nitrate (10^−2^ M) with a mixture of 90 ml composed of hydroxylamine hydrochloride (10^−3^ M) and sodium hydroxide (10^−3^ M). The AgNps were obtained at room temperature under rapid stirring conditions and showed an extinction maximum at 407 nm (S2 Fig).

Textile samples were coated with a concentrated paste of gold or silver nanostructures. The pastes of Au and Ag nanoparticles were obtained by centrifugation to >10.000 g of 1 mL aliquots of colloidal solution.

The following reagents were used: tetrachloroauric (III) acid (≥99.9% trace metals basis), silver nitrate (99.9999% trace metals basis), trisodium citrate dihydrate tribasic ≥99.0%, hydroxylamine hydrochloride (99.999% trace metals basis), all from Sigma-Aldrich, sodium hydroxide (max. 0.002% K, ACS, Reag. Ph Eur) from Merck. Ultrapure type 1 water (18.2 MΩ.cm at 25 °C, TOC ≤ 5 ppb) was employed for all procedures. All of these chemicals were applied as received.

***2.2.6.1. Analysis of archaeological textiles*:** SERS spectra of the samples were recorded with a Renishaw InVia Reflex Raman apparatus equipped with 532, 633, and 785 nm laser lines, a Leica microscope, and an electrically cooled CCD detector [5]. The instrument was calibrated using the 520 cm^−1^ line of a Si plate and a 50X objective. This equipment has a 4 cm^−1^ resolution, and 1−10 scans of 10−50 s each were averaged. Spectra are presented in the 2000−200 cm^−1^ region. Laser powers between 10 and 100 mW were used. Spectral scanning conditions were chosen to avoid photodegradation and photodecomposition of the sample. Only the 785 nm laser line was used for all measurements. Data was collected, processed, and graphed using WIRE 3.4, GRAMS 9.0, OMNIC 9.2.98, and OriginLab Pro 2016 programs. SERS spectra were baseline-corrected using the automatic baseline option of the software OMNIC, and then smoothed via a Savitzky–Golay algorithm, using 30 points and a second-grade polynomial. At least five SERS spectra were acquired on each fiber and then averaged.

***2.2.6.2. Analysis of undyed wool and a modern textile dyed with shellfish purple*:** Raman measurements were acquired with a portable i-Raman EX spectrometer (B&W Tek), with a Nd-YAG 1064 nm laser, and an InGaAs array detector with 2500–175 cm^−1^ spectral range and 9.5 cm^−1^ resolution. In order to avoid laser induced damage, power was set to no more than 180 mW. Acquisition times ranged from 5 s to 60 s per scan, and 3–10 spectra were averaged on each scan.

SERS data was collected with two i-Raman Plus from BW&TEK, with 532 nm and 785 nm laser sources. Laser power was set at 37 mW, with a 4000–175 cm^−1^ spectral range and a 4 cm^−1^ resolution. Acquisition time also varied from 5 to 30 s, with 3–10 averages per point. All three spectrometer detectors employ an array of CCD.

#### 2.2.7. Fiber-optics Reflectance Spectroscopy (FORS).

FORS was applied to confirm the presence of dibromoindigo on a modern textile (S13 Fig). Spectra were acquired with an ASD FieldSpec-4 device (Malvern Panalytical, UK), in the 350–2500 nm spectral range. The equipment was used in contact mode with a CIE D65-series light source. The analyzed area is approximately 3 mm dia. and 25 spectra were averaged over 5 s. Calibration was performed using a certified reflectance standard (AS-02035-000CSTM-SRM-990-362, ASD Inc.).

## 3. Results and discussion

### 3.1. Fiber identification

**Fig 4:**
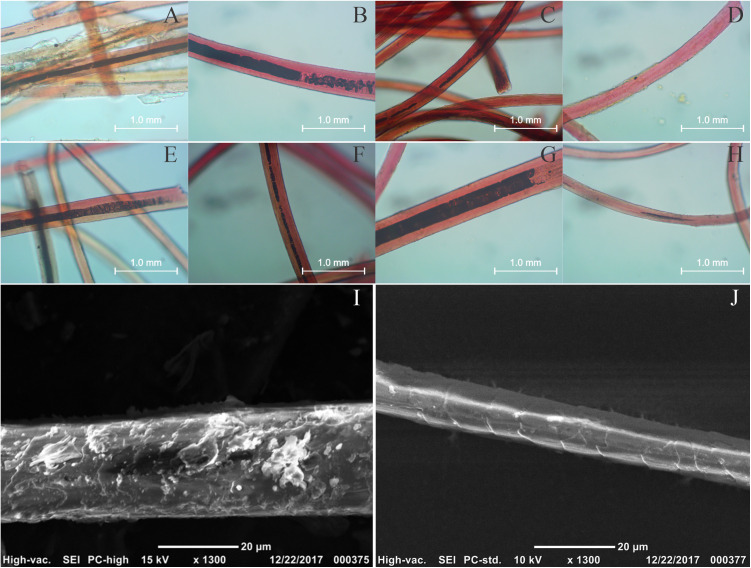
40X optical microscopy images of unwashed fibers 799 (A), 804 (B), 806 (C) and 807 (D) and washed fibers 799 (E), 804 (F), 806 (G) and 807 (H); SEM images of unwashed (I) and washed (J) fiber 799.

Fig 4A–4H show microscopic images of the washed and unwashed textiles obtained with 40X magnifications. These images show anatomical features of animal threads, keratin medulla and cortex are observed for all fibers [5,43]. More detailed morphological images of these materials were acquired via SEM (Fig 4I and 4J). Here, the characteristic scales of animal fibers are clearly observed [50–52], although this morphological information is often insufficient to discriminate among the fibers of different camelid species [51,53], a common source of animal threads in the Andean region.

**Fig 5:**
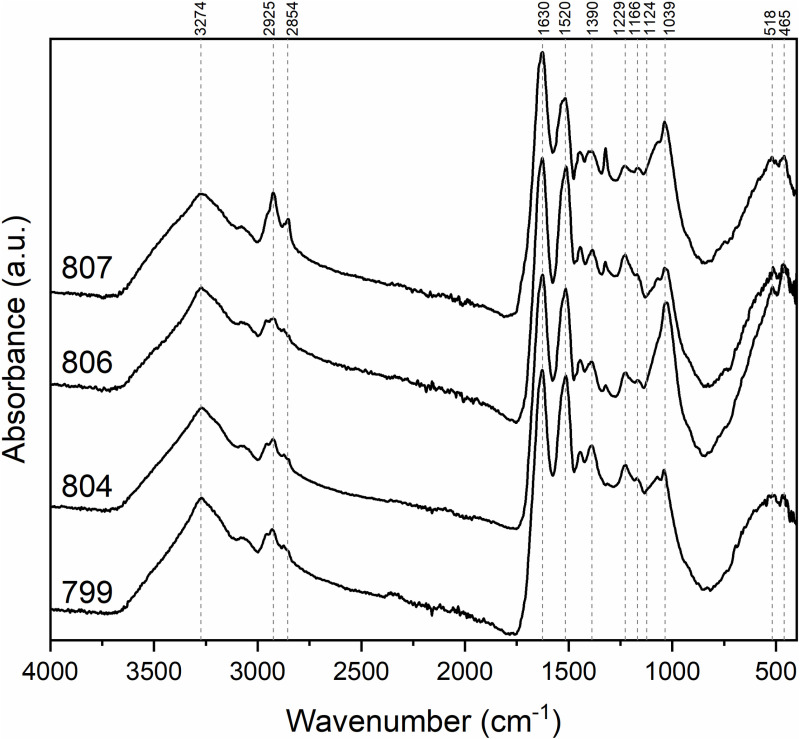
Infrared spectra of the washed fibers.

A recent paper by Quispe suggests that such discrimination can be successfully achieved through FTIR analysis [54]. They studied South American camelid and goat fibers and found that a set of bands in the 1200–1000 cm^−1^ region can be used as markers, in particular the differences in their intensities. In our case, the FTIR spectra of the washed fibers (Fig 5) present a medium to intense band around 1038 cm^−1^, which along with the absence of the band at 1124 cm^−1^ and the low intensity of the band at 1166 cm^−1^ points to the use of *vicuña* fibers. The spectral profile of both washed and unwashed fibers showed little variation, indicating that the protein’s structure remains unchanged after the washing protocol. A detailed description of the bands observed in the FTIR spectra is provided in the supplementary material (Suppl. 3. FTIR measurements in S1 File).

### 3.2. XRF analyses

**Fig 6:**
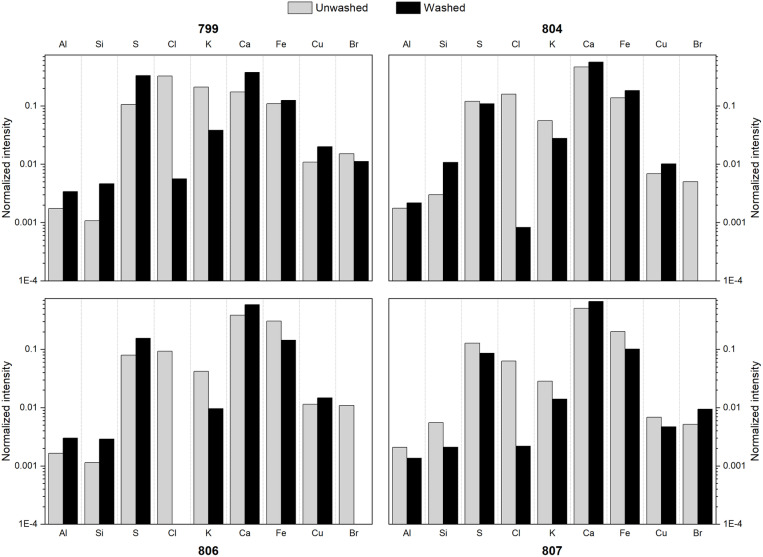
Normalized elemental X-ray intensities from XRF analyses of the unwashed (grey bars) and washed (black bars) samples.

The soil’s elemental profile, obtained by *in situ* XRF analysis at the PLM-3 archaeological site, was consistent with sandy and saline soil, with high Fe, Ca, and Cl contents (See Tables S1 and S2 in S1 File in the Supplementary material). Most elements observed in the soils at PLM-3 are also present in the unwashed fiber samples and generally follow a similar trend. However, elemental content changed when analyzing the washed fibers, particularly for Al, Si, S, Cl, K, Ca, Fe, and Br (Fig 6). Notably, the amount of Cl is greatly diminished on the washed fibers -and potassium to a lesser extent-, confirming that the contamination related to seawater salts is considerably reduced. This result is in accordance with the SEM images of both unwashed and washed fiber 799 (Fig 4E and 4F), where the unwashed fiber showed particles adhered to the fiber, apparently composed of heavier elements, from the contrast observed on the backscattered electrons image. Such particles are absent on the SEM image of the washed fiber. In contrast, only minor changes were observed in the Si content, indicating that the washing protocol was not effective in removing the sand sediments.

A direct comparison between the elemental content of the soil and the fibers is challenging due to the differences in the analytical conditions used, including the use of two spectrometers and the nature of the samples analyzed. To partially help address this problem, a NIST’s Montana soil reference (SRM 2711) was also measured with both X-ray spectrometers under the experimental conditions described previously to demonstrate that the set of detected elements is essentially the same (S3 Fig) and the spectra X-ray normalized intensities can be compared.

Many of the elements detected could be related to the dyeing process (Al, S, K, Ca, Fe, and Cu). Aluminum, potassium, iron, and copper are part of the composition of the most commonly employed mordanting salts (K_2_O_4_Al_2_(SO_4_)_3_, FeSO_4_·7H_2_O and CuSO_4_·5H_2_O) [18,21,55]. Nevertheless, it is important to note that the sediment contamination of the samples did not allow us to clearly distinguish the presence of these elements on the fibers themselves. Further studies on the fibers would be required to determine the use of a particular mordanting salt.

In addition, the presence of sulfur on the washed fibers could be related to the sulfate anions from the mordants and the cysteine bridges that are abundant in proteinaceous fibers [18], including camelid fibers. Calcium was also detected, potentially related to the alkaline conditions necessary for improved solubility of certain dyes in water. Calcium was previously reported in Paracas textiles dyed with indigoids [18], which is consistent with one of our findings.

Finally, the presence of bromine in all four unwashed fibers is interesting. It could be related with the contamination from marine salts, the tomb’s soil, or to the use of a brominated dye. Bromine was not detected in the p-XRF analysis of the soil mentioned above, thus ruling out the hypothesis of transference from the adjacent soil. After washing, the bromine content is maintained in fibers 799 and 807 (Fig 6). Hence, the presence of bromine in these two samples is likely unrelated to marine salt contamination.

The shades of some red-purple areas in *inkuñas* 799 and 807, together with the presence of bromine, suggest that brominated derivatives of indigoids (monobromo-indigo, dibromoindigo, or dibromo-indirubin) were used in the dyeing process [56]. The precise identification of these indigoid dyes requires a molecular analysis, which will be further addressed in the discussion of the SERS results.

### 3.3. SERS measurements

**Fig 7:**
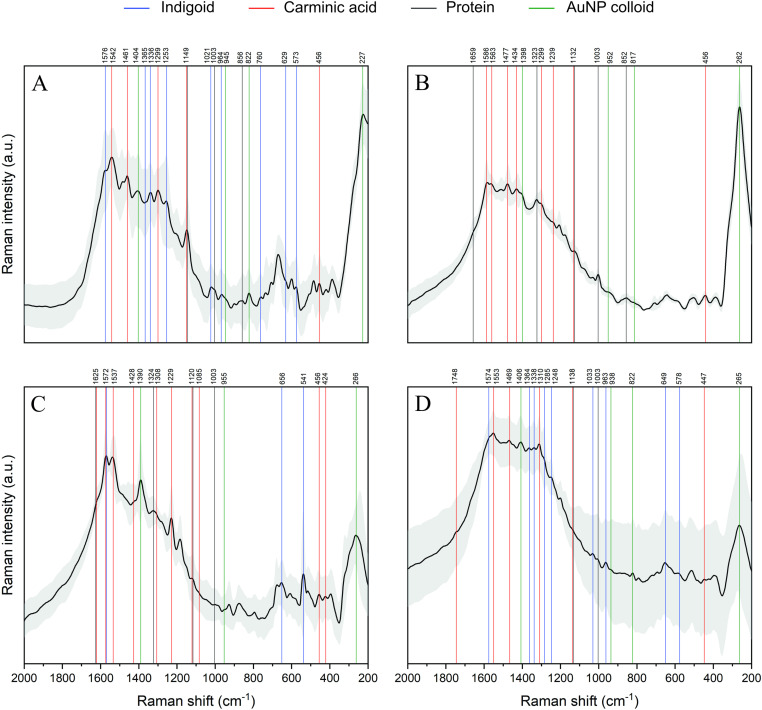
Average SERS spectrum (black line) and standard deviation (gray area) of fibers 799 (A), 804(B), 806 (C) and 807 (D).

The results of SERS analysis on the four dyed fibers are shown in Fig 7A–7D, where the average SERS spectrum for each fiber is shown, along with its standard deviation. Each averaged spectrum includes the information of five (six for textile 807) individual spectra. These individual spectra are shown in Figs S8–S11 in S1 File (Supplementary material), and the detailed list of identified bands and its vibrational assignment is presented in Table 1. For comparison purposes, SERS spectra of an undyed wool fiber were acquired using similar gold colloids and two laser beams, 532 and 785 nm, together with the spectra of the colloids (see Fig S12 in S1 File, Supplementary material). No clear Raman signals were observed in any of these last four cases, except for those related to the Au-O vibrations at low wavenumbers.

**Table 1:** Raman bands identified for each fiber. Renishaw InVia Reflex Raman 785 nm laser, Au colloid.

Fiber 799	Fiber 804	Fiber 806	Fiber 807	Assignment	
			1748	ν_acid_(C = O)	Carminic acid
1666 − 1640	1659	1625		Amide I/ νCO/ νCC	Protein / Carminic acid
	1586	1572		ν(CC)/ δ(C_5_OH)/ δ(CH)	Carminic acid
1576		1572	1574	νC = C/ νC = O	Indigoid
1542	1563	1537	1553	ν(CC)/ δ(C_8_OH)/ δ(CH)	Carminic acid
1461	1477		1469	ipδCH_3_/ ipδCOH/ νCC/ δCH	Carminic acid
	1434	1428		ν(CC)/ δ(CH_3_)/ δ_Glu_(CH)	Carminic acid
1404	1398	1390	1406	ν_asym_COO	Colloid
1365			1364	δ(NH)/ δ(CH)	Indigoid
1336			1338	ρ(NH-C = C-NH)	Indigoid
	1323	1324		Amide III	Protein
1299	1299	1308	1310	δ(C_5_OH)/ δ(C_8_OH)/ δ(C_3_OH)/ ν(CC)	Carminic acid
			1285	νCC_ring_	Indigoid
1253			1248	δNH/ δC = O	Indigoid
	1239	1229		δCH/ δCCC; δCH δ(C_5_OH)/ δ(C_4_H)/ ν(CC)/ δ_Glu_(CH)	Carminic acid
1149	1132	1120	1138	δNCH Pro/ ν(CC)/ δ(COH)/ δ(CH)	Protein/ Carminic acid
		1085		ν_Glu_(CO)/δ_Glu_(COH)	Carminic acid
1021			1033	δCH	Indigoid
1003	1003	1003	1003	Breathing of aromatic ring Phe	Protein
964			963	δCH	Indigoid
945	952	955	938	νC–COO	Colloid
856	852			Skeletal vibration	Protein
822	817		822	νCCCC-O	Colloid
760				δ(CH)/ δ(NCC)	Indigoid
629		656	649	γ(NH)	Indigoid
573		541	578	δ(CH)/ δ(NCC)	Indigoid
456	456	456	447	Skeletal vibration	Carminic acid
		424		Skeletal vibration	Carminic acid
305				Br-C stretching	Indigoid
227	262	266	265	Au-O stretching	Colloid

The averaged SERS spectrum of fiber 799 (Fig 7A) displays bands that can be attributed to the gold nanoparticle colloid used to enhance the Raman signal, to the keratin protein, to carminic acid and to an indigoid dye. Previous studies on citrate-reduced gold nanoparticles report bands related to the asymmetric stretching of the –COO group around the 1404 cm^−1^, the stretch modes of C–COO and CCCC–O at 945 and 822 cm^−1^ and Au-O stretching at 277 cm^−1^ [57–59].

The presence of the protein is inferred by a set of bands associated with the α-helix secondary structure of keratin, mainly amide I, amide III, CC skeleton, and residues of the amino acids phenylalanine and proline [4,54]. In fiber 799 (Fig 7A), amide I is observed as a weak shoulder in the 1670−1600 cm^−1^ region, a δNCH Pro vibration at 1149 cm^−1^, and the CC skeleton vibration at 857 cm^−1^. The band around 1000 cm^−1^ is attributed to an oblate-prolate or breathing vibration mode of the aromatic rings [5,60]. This vibration is characteristic of the amino acid residue phenylalanine which has been widely reported in proteins [5,60].

Five bands from the average spectrum in Fig 7A can be linked to the presence of carminic acid, while a sixth band is present in two of the individual spectra presented in S8 Fig. This last band, a shoulder in the 1666−1640 cm^−1^ region, is attributed to coupled νCO/νCC vibrational modes [31–33,61,62], and is probably overlapped with the amide I band of the proteinaceous fiber. The band at 1542 cm^−1^ can be assigned to a νCC_ring_ vibration, coupled with in-plane bending of C-OH and C-H, and that at 1461 cm^−1^ to a combination of ipδCH_3_/ipδCOH/νCC and δCH vibrational modes. A medium intensity band at 1299 cm^−1^ has been widely reported [5,11,61] and is related to a stretching deformation mode of a 6-membered ring and in-plane C-OH bending. The most intense band identified in our spectrum, located at 1149 cm^−1^, is attributed to the ν(CC)/δ(COH)/δ(CH) coupled mode. The reported data show that this band can be found in the 1160–1130 cm^−1^ range, with low intensity [5,61]. This band is also overlapped with the Proline band reported above. Finally, the band at 456 cm^−1^ can be attributed to a skeletal vibration.

The last set of bands, marked in blue on Fig 7A, may correspond to an indigoid dye. This is supported by the characteristic band at 1576 cm^−1^ observed in the average spectrum [5,10,30,63–69]. This signal, widely reported in the literature, is attributed to a combination of CC and CO stretching (νCC/νCO) modes and, although observed as a weak band in Fig 7A, is present as an intense band in two of the individual spectra shown in S8 Fig. Derivatives of dibromoindigo have been reported to show a particular set of vibrational signals in the region between 1230 and 1365 cm^−1^ [30]. The intensity of these bands varies depending on the indigoid derivative in question [30,63–69]. For instance, in indigotin, the signal around 1365 cm^−1^ is of high intensity, in 6, 6’-dibromoindigo, the most intense band is centered at 1253 cm^−1^, while in 5,5`-dibromoindigo three bands of similar intensity are located at 1345, 1278 and 1239, cm^−1^ [30]. In our case, three bands are observed at 1365, 1336, and 1253 cm^−1^ in the spectrum of sample 799 (Fig 7A), although the band at 1365 cm^−1^ is very weak on the average spectrum, only displaying a medium intensity in one of the individual spectra from S8 Fig. These vibrational signals are assigned to the δ(NH)/δ(CH), ρ(NH-C = C-NH), and δNH/δCO modes, correspondingly.

Additional dibromoindigo bands at 1021 and 964 cm^−1^ (Fig 7A) are attributed to the deformation mode of the CH fragment (δCH) [63–65] and the combined vibration mode νCH/δCC of rings, respectively [64,65]. Other bands are observed at 760, 629 and 573 cm^−1^, and are related to δ(CH)/δ(NCC), γ(NH) and δ(CH)/δ(NCC), respectively [30,64]. Finally, a weak band near 305 cm^−1^ is visible as a shoulder in two of the individual spectra of S8 Fig and has been related with the Br-C stretching mode of dibromoindigo by Karapanayiotis [64]. As mentioned above, XRF analysis of the pre and post-washed fiber allowed us to detect the consistent presence of bromine, supporting the identification of a brominated indigoid derivative.

A similar analysis was performed for the remaining fibers (Figs 7B–7D, S9–S11). The average spectrum of fiber 804 (Fig 7B) presented bands related to the gold colloid (1398, 952, 817 and 262 cm^−1^), to a proteinaceous fiber (1659, 1323, 1132, 1003 and 852 cm^−1^) and to carminic acid (1586, 1563, 1477, 1434, 1299, 1239, 1132 and 456 cm^−1^).

An almost similar case was observed for the fiber 806 (Fig 7C), where most of the identified bands correspond to the nanoparticles colloid (1390, 955 and 266 cm^−1^), the fiber itself (1625, 1324, 1120 and 1003 cm^−1^) and carminic acid (1625, 1572, 1537, 1428, 1308, 1229, 1120, 1085, 456 and 424 cm^−1^). However, three distinct indigoid bands are clearly visible in one of the individual spectra of S10 Fig, at 1572, 656 and 541 cm^−1^. These three bands show medium to high intensity and, while only present in one of the five individual spectra, are still clearly visible in the averaged spectrum (Fig 7C). All three bands have been reported specifically for indigo [10,65,70–72]. The absence of bromine in the XRF results of the washed fibers supports the identification of indigo, instead of a brominated indigoid derivative.

The average spectrum of fiber 807, presented in Fig 7D, showed a lower signal to noise ratio – when compared to the previous three – and a larger standard deviation. Again, four sets of bands were identified: those corresponding to the citrate-reduced gold nanoparticles at 1406, 938, 822 and 265 cm^−1^, a second set related to the textile fiber at 1138 and 1003 cm^−1^, carminic acid bands at 1748, 1553, 1469, 1310, 1138 and 447 cm^−1^ and, finally, indigoid bands – probably related to dibromoindigo [30,63–69] – at 1574, 1364, 1338, 1285, 1248, 1033, 963, 649 and 578 cm^−1^. As in the previous cases, these bands are more clearly distinguishable in some of the individual spectra from S11 Fig. As for fiber 799, XRF analysis of fiber 807 showed the presence of bromine after washing.

Given the relevance of the proposed identification of dibromoindigo, further studies were performed to support the findings by SERS measurements. Unfortunately, the authors were unable to perform HPLC – the most common technique employed in dye identification – and/or FORS studies when the archaeological fibers were available for analysis. Nevertheless, a modern textile dyed with shellfish purple (S13 Fig) was available to reinforce the proposed identification of a brominated indigoid. This modern textile is part of the reference material collection of LANCIC (UNAM, Mexico) and both the presence of dibromoindigo in the purple areas of the textile, as well as the vegetable nature of the fibers, were confirmed via FORS (S14 Fig) [63,73–76].

Four sets of studies were performed on the textile: XRF, Raman with a 1064 laser beam and SERS with citrate-reduced Au nanoparticles and with hydroxylamine-reduced Ag nanoparticles. XRF spectra were collected at a reddish and a purple area of the textile and are presented on S15 Fig. The characteristic K bromine peaks are clearly visible (Kα at 11.9 keV) in the spectra acquired from the purple area, while being absent in those acquired from the reddish area.

**Fig 8:**
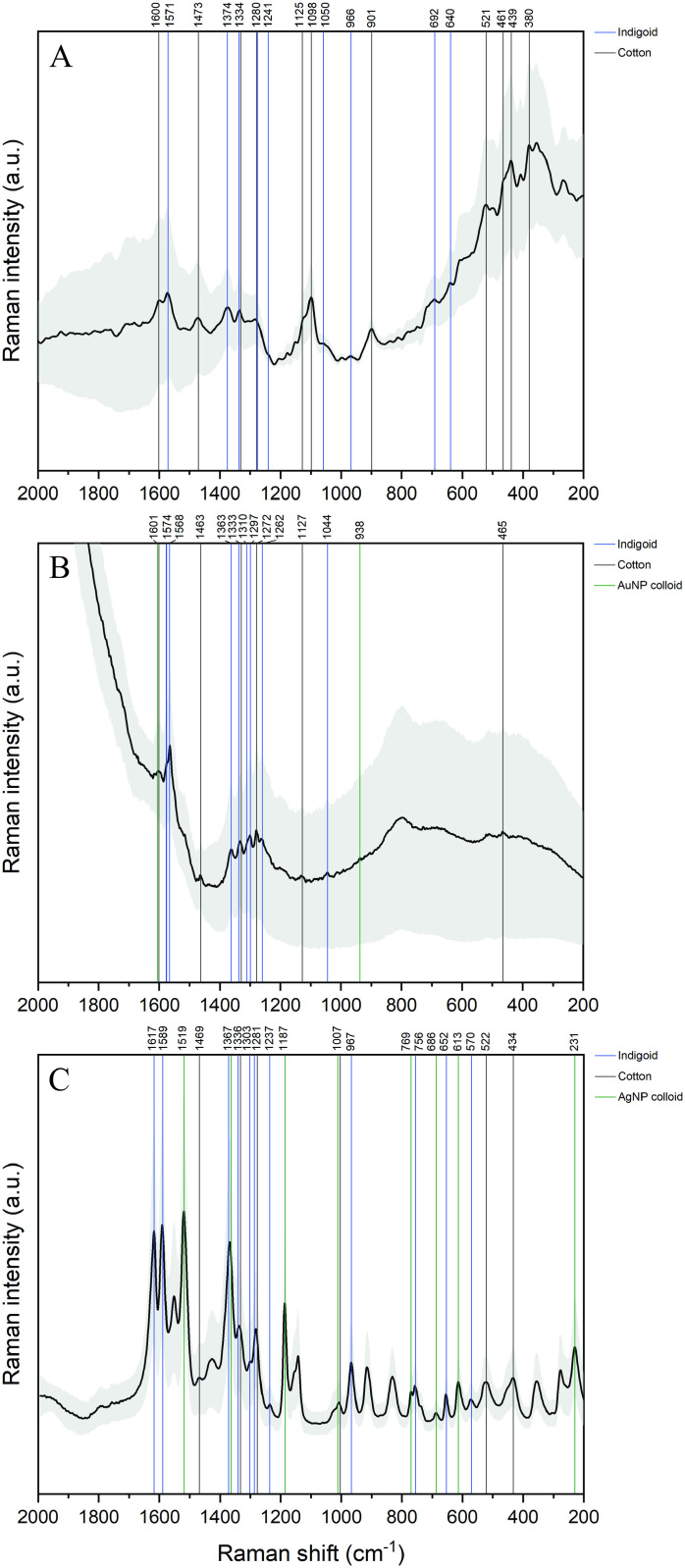
Average spectra (black line) and standard deviation (grey area) of a purple fiber from a modern textile dyed with shellfish purple. **A)** 1064 nm Raman; **B)** Au nanoparticles SERS; **C)** Ag nanoparticles SERS.

The averaged 1064 nm Raman spectrum of the purple area (Fig 8A) shows cotton-related bands at 1600, 1473, 1334, 1280, 1125, 1098, 901, 521, 439, 380 cm^−1^ [77,78]. In addition, nine dibromoindigo bands are observed; an intense band at 1571 cm^−1^ and three medium bands at 1374, 1334 and 1280 cm^−1^, the last two overlapping with a cotton band [30,63–69]. A shoulder at 1241 cm^−1^, previously reported by Smith for 5,5-dibromoindigo [30], and three weak bands at 966, 692 and 640 cm^−1^ can also be observed.

The SERS spectra of purple fibers sampled from the textile also showed bands corresponding to the nanoparticle colloid, the cotton fiber and the dye. First, we used citrate-reduced gold nanoparticles, prepared in the same manner as those applied to the archaeological fibers. In Fig 8B, the colloid bands are hardly visible as two weak signals at 1061 and 938 cm^−1^ and the cotton bands are present as two medium-intensity signals at 1333 and 1272 cm^−1^, and three weak bands at 1463, 1127 and 465 cm^−1^. Indigoid-related bands are present as a shoulder and an intense signal at 1574 and 1568 cm^−1^, a group of medium bands in the 1363–1262 cm^−1^ region, and a weak band at 1044 cm^−1^.

Finally, the suitability of SERS for the detection of dibromoindigo on a dyed textile was further confirmed by the application of silver nanoparticles to a second purble fiber sampled from the modern textile (Fig 8C). Here, a considerable number of well-defined bands are observed, including colloid bands at 1519, 1367, 1187, 1007, 769, 686, 613 and 231 cm^−1^ [79], and cotton bands at 1469, 1336, 1281, 1007, 522 and 434 cm^−1^. Furthermore, up to 11 indigoid bands were detected: two very intense bands at 1617 and 1589 cm^−1^, three bands overlapped with colloid or cotton bands at 1367, 1336 and 1281 cm^−1^, two weak bands at 1303 and 1237 cm^−1^, and four bands at 967, 756, 652 and 570 cm^−1^.

Although differences in dyeing conditions between the modern textile and the archaeological fibers, as well as the weathering and ageing processes the latter underwent could lead to small discrepancies in Raman band positions, most of indigoid-related bands are observed at the same wavenumber or at a very close value (± 10 cm^−1^). This displacement has already been observed for the Raman and SERS spectra of cochineal at different pH levels [61].

Overall, XRF, Raman and SERS data from the modern textile support the proposed identification of an indigoid dye, most likely dibromoindigo, in two of the archaeological fibers analyzed. Firstly, bromine XRF lines were only observed in the spectra of the areas dyed with shellfish purple on the modern textile, confirming that this element is a first indication of the presence of shellfish purple both on this textile and on the archaeological fibers. Secondly, the Raman and SERS spectra acquired by three different methods – one of them using gold nanoparticles prepared under the same procedure as those used for the archaeological samples – on fibers from the modern textile consistently display bands associated with a brominated indigoid, which have been reported in the available literature [30,63–69].

The successful application of Raman and SERS for the identification of dibromoindigo on the modern textile confirms the suitability of both techniques for this purpose. Even with the absence of HPLC, which is a shortcoming in our study, these tests on the modern textile support the proposed identification of a brominated indigoid derivative in archaeological fibers 799 and 807.

### 3.4. Discussion

Results obtained in the present study are synthesized in Table 2 and demonstrate similar materials and technologies, which are characteristics of a shared cultural tradition of textile production on the northern coast of Chile during pre-Hispanic times.

**Table 2:** Synthesis of results obtained in the present study.

Fiber	Color	Origin of fiber via OM, SEM and FTIR	Dye identification with SERS
799	Red tone	Animal, vicuña*	Cochineal, shellfish purple*
804	Dark red tone	Animal, vicuña*	Cochineal
806	Dark red tone	Animal, vicuña*	Cochineal, indigo*
807	Dark red tone	Animal, vicuña*	Cochineal, shellfish purple*

* Proposed identification.

Elemental analysis by XRF identified the presence of elements that could be related with mordants reported in other Andean textile traditions (mainly K_2_O_4_Al_2_(SO_4_)_3_, FeSO_4_·7H_2_O and CuSO_4_·5H_2_O) [19]. However, the low concentration of mordanting salts in textiles, compared to the high amount of archaeological sediment contamination, prevented us from clearly associating the detection of these elements with the use of a specific mordant. This must be the subject of further research, which should include a detailed elemental mapping via SEM-EDS of the surface of the fibers. Nonetheless, XRF analysis did allow us to identify bromine in two of the samples, which was associated with the use of a brominated dye.

The combination of optical microscopy (OM), scanning electron microscopy (SEM) and FTIR-ATR led to the confirmation of the use of animal fibers. Animal fibers were extensively employed in the late pre-Columbian periods, and the use of camelid fibers has been recognized since the Archaic period, around 3700 BC, by the coastal Chinchorro cultural tradition [4]. FTIR results also provide an indication of the particular camelid species from which the fibers were obtained, *vicuña*, according to recent research on South America camelids via FTIR-ATR [54].

As previously indicated, the use of SERS to identify dyes in archaeological and heritage samples has had some success, [5,11,30–34], although HPLC remains the most accurate method for this purpose and should be applied whenever possible. Sepúlveda and collaborators used AuNps and a 785 nm laser beam [5], while Celis and co-authors used AgNps and a 532 nm laser [11]. Both identified carminic acid as a dye in all the textile samples belonging to funerary contexts from around 1100 BC to 1400 AD. Sepúlveda and co-authors identified the presence of indigo and indigoids since the Formative period (around 1.100 B.C.- 550 A.D) [5]. Our results point to a new combination of carminic acid and dibromoindigo in the Andean region.

The earliest reported use of carminic acid in the area – extracted from cochineal – was around 1100 BC in the inland region of the northern Atacama Desert, which was earlier than the Central Andes region of Peru [5]. The known natural sources of dibromoindigo are specific shellfish species distributed worldwide [30,64]. Three of them have been reported to inhabit the Pacific coast of South America: Red-mouthed rock shell of the Eastern Pacific (*Stramonita biserialis*) from Mexico to Chile; *Loco* (*Concholepas concholepas*) from Callao, near Lima, Peru, to the Strait of Magellan in the southern end of Chile, including the Juan Fernández Archipelago, and Chocolate rock shell (*Stramonita chocolata* or *Thais Chocolata*) from Peru to Valparaiso, Chile [80–82]. In the north of Chile, these shellfish have been exploited since the Archaic period – around Cal. 7500 BC – for food consumption and the shells as containers or instruments [83]. Our proposed identification of shellfish purple dye suggests that textile producers combined it with another dye, obtaining a red-purple tone from this dye mixture. It reaffirms the close interactions of the population of PLM-3 with the sea and the deep knowledge derived from the shell collection.

Regarding the use of this dye for textile production, Phipps [84] indicated that dyed purple yarns appeared no later than 500 AD. Other Andean cultural traditions employed purple-dyed fibers with red dyestuff, overdyed with indigo. In 1963, Saltzman and collaborators found shellfish purple dye in pre-Hispanic cloth [85], and Michel [86] identified it on a fabric from Pachacamac, Peru, dated back to 900–1200 AD. Other authors have found that purple dyes could be prepared from a mixture of cochineal and indigo as identified in Mexico [87], while a combination of cochineal and *maíz morado* (purple corn) was found in Peru [88]. In 2016, a complex mixture of dyes was observed in textiles from Nasca, an ancient cultural tradition in Peru, but the individual dyes could not be identified [89]. The use of specific mordants could contribute to modifying the red shade and obtaining purple to dye yarns [87,88].

Thus, the proposed identification of a dye mixture could expand our knowledge of textile production in the Andean region. The variety of red tones and colors obtained with several dyes in *Inkuña* textiles indicated the absence of a specific norm for their production and the possibility of innovation. In the future, we must delve deeper into the origin of shellfish dyeing practice and its antiquity on the coast north of the Atacama Desert in Chile. Numerous *inkuñas* with different dyeing processes in the same tomb revealed a greater complexity associated with the textile production of the inhabitants of this region.

## 4. Conclusions

A non-invasive, multitechnique methodology was successfully applied for the first time to analyze millimeter-size textile samples from Playa Miller-3 archaeological site (1100–1450 AD), located on the coast of the Atacama Desert in northern Chile. This study expands our knowledge on pre-Columbian textiles and their production techniques, while highlighting the benefits of applying a multi-analytical approach to small samples.

The macro and microscopic techniques allowed us to identify different shades of red, the presence of solids adhered to the surface of the samples, and the fibrillar structure of animal fibers. Our experience working with archaeological samples from northern Chile also led us to develop a washing procedure for textile samples using a non-ionic detergent (with dilute surfactant Triton X100), which effectively removed most seawater residues.

Regarding the spectroscopic techniques, elemental analysis by XRF did not allow us to clearly identify any mordants on the samples. However, this technique detected the presence of sulfur, probably related to animal fibers, and bromine, associated with the use of a brominated indigoid dye. Optical microscopy, SEM and FTIR confirmed the use of animal fibers, likely from *vicuñas*. Finally, this study demonstrated the advantages of using gold nanostructures in SERS analyses for the identification of the dyes used. The application of this protocol suggested the use of animal fibers dyed with carminic acid and indigoids. Further studies by HPLC would be required to fully confirm these results.

In addition to confirming the use of carminic acid as the primary dye for red fibers in pre-Columbian and coastal archaeological textiles, we proposed the use of a dye mixture composed of cochineal (carminic acid) and shellfish purple (dibromoindigo). Mollusks were also collected for dyeing purposes on the north coast of the Atacama Desert. Our findings provide new insights into the millennial textile production practices of the region’s inhabitants, particularly into the technologies used to produce *inkuñas* during the Late Intermediate period (1100–1450 AD), showcasing their accumulated knowledge and interaction with animals, plants and shellfish.

## Supporting information

S1 File Supplementary materials. Docx document containing all supplementary text, figures, tables and references.
(DOCX)

S1 Fig A) Playa Miller 3 (PLM-3) near the Pacific Ocean; B) Two transects followed for XRF in situ analysis at Playa Miller-3 archaeological site (drone image). (TIF)

S2 Fig UV-Vis absorbance spectra of the gold and silver colloids. (TIF)

S3 Fig XRF spectra of the NIST SRM 2711 Montana soil. Comparison of detected elements under the experimental conditions described in the experimental section for SANDRA and Bruker Tracer III-SD X-ray spectrometers.
(TIF)

S4 Fig FTIR-ATR spectra of sample 799, (A) unwashed and (B) washed. (TIF)

S5 Fig FTIR-ATR spectra of sample 804, (A) unwashed and (B) washed. (TIF)

S6 Fig FTIR-ATR spectra of sample 806, (A) unwashed and (B) washed. (TIF)

S7 Fig FTIR-ATR spectra of sample 807, (A) unwashed and (B) washed. (TIF)

S8 Fig SERS spectra of fibre 799. (TIF)

S9 Fig SERS spectra of fibre 804. (TIF)

S10 Fig SERS spectra of fibre 806. (TIF)

S11 Fig SERS spectra of fibre 807. (TIF)

S12 Fig 532 nm SERS spectra of an undyed wool fibre using gold nanoparticles (A) and the gold colloid (B); 785 nm SERS spectra of an undyed wool fibre using gold nanoparticles (C) and the gold colloid (D). (TIF)

S13 Fig Modern textile dyed with shellfish purple (purple areas). (TIF)

S14 Fig FORS spectra from the red and purple areas of a modern cotton textile. Purple areas were dyed with shellfish purple.
(TIF)

S15 Fig XRF spectra from the red and purple areas of a modern cotton textile. Purple areas were dyed with shellfish purple.
(TIF)

S1 Table XRF elemental peak intensities (counts) of the *in situ* soil analyses at PLM-3. Intensities correspond to K lines (Al - Mn).
(DOCX)

S2 Table XRF elemental peak intensities (counts) of the *in situ* soil analyses at PLM-3. Intensities correspond to K lines (Fe - Zr).
(DOCX)
