# Supplementary material for: New insights into archaeological textiles (1000–1450AD) from the coastal region of the Atacama Desert: Preliminary evidence of a cochineal and shellfish purple dye combination
Source: PLoS One. 2025 Jun 4;20(6):e0325623. doi: 10.1371/journal.pone.0325623 (PMC12136422; doi:10.1371/journal.pone.0325623)
Supplement: S1 File — Docx document containing all supplementary text, figures, tables and references. (DOCX) [file pone.0325623.s001.docx]

**Supplementary Materials**

***Suppl. 1. Methods***

**
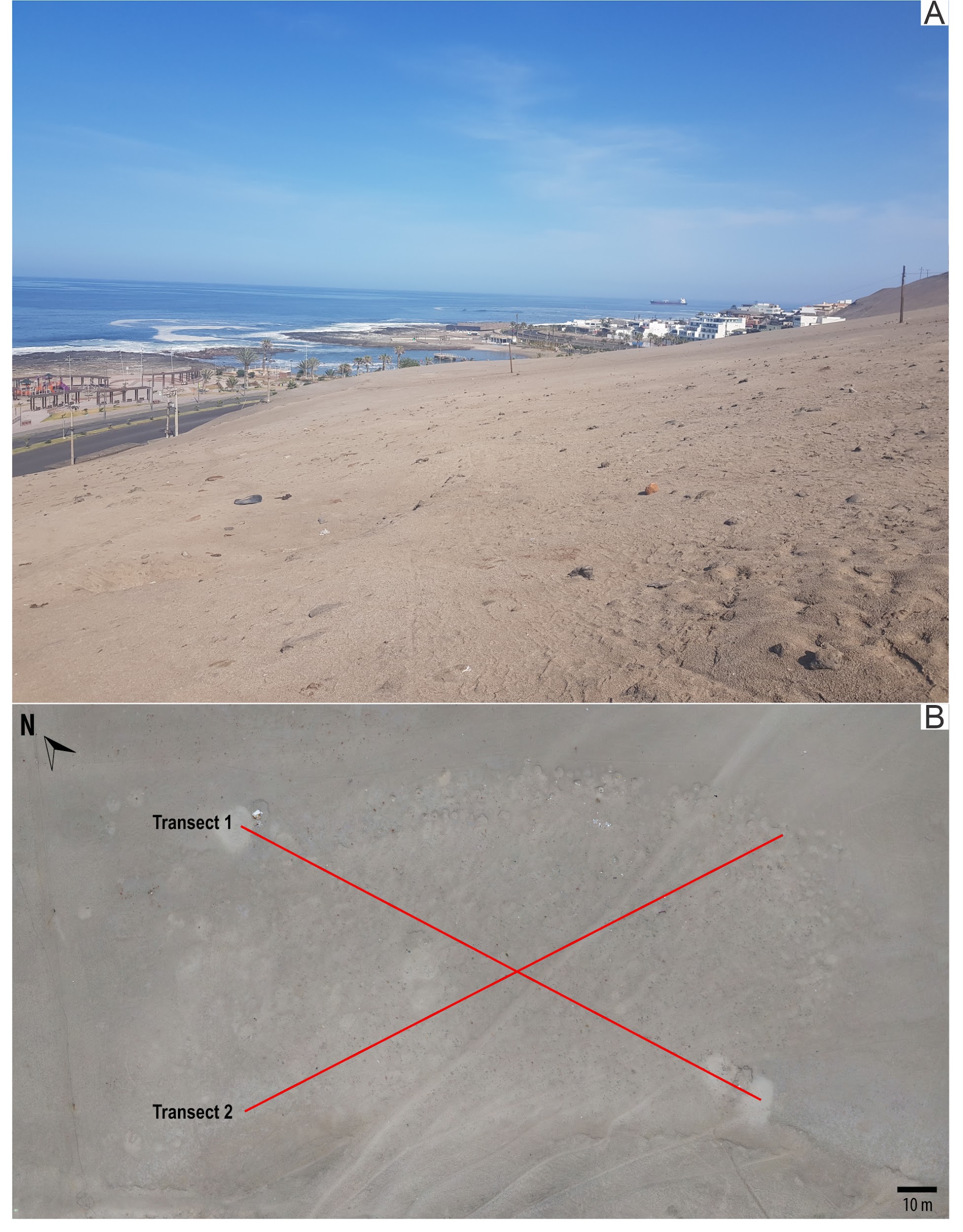
**

**Fig S1.** *A) Playa Miller 3 (PLM-3) near the Pacific Ocean; B) Two transects followed for XRF in situ analysis at Playa Miller-3 archaeological site (drone image).*
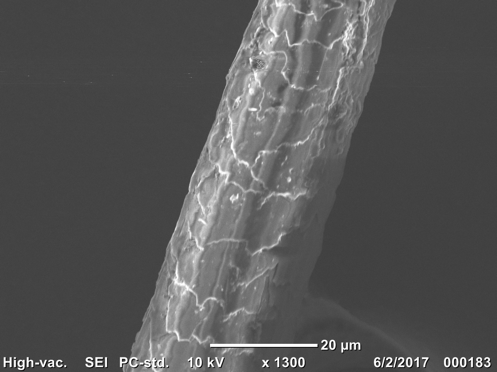


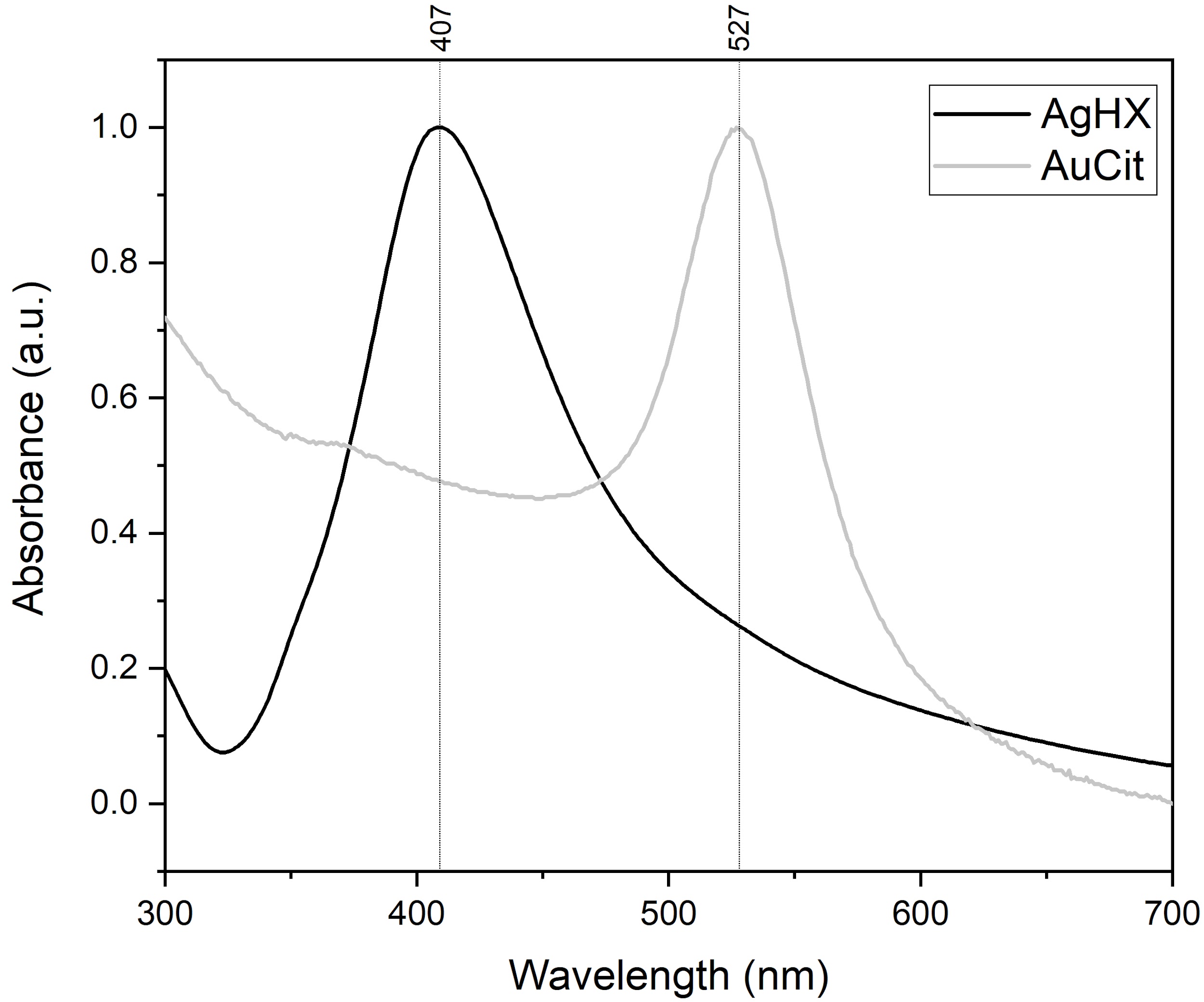


**Figure S2.** UV-Vis absorbance spectra of the gold and silver colloids

***Suppl. 2. XRF measurements***

**
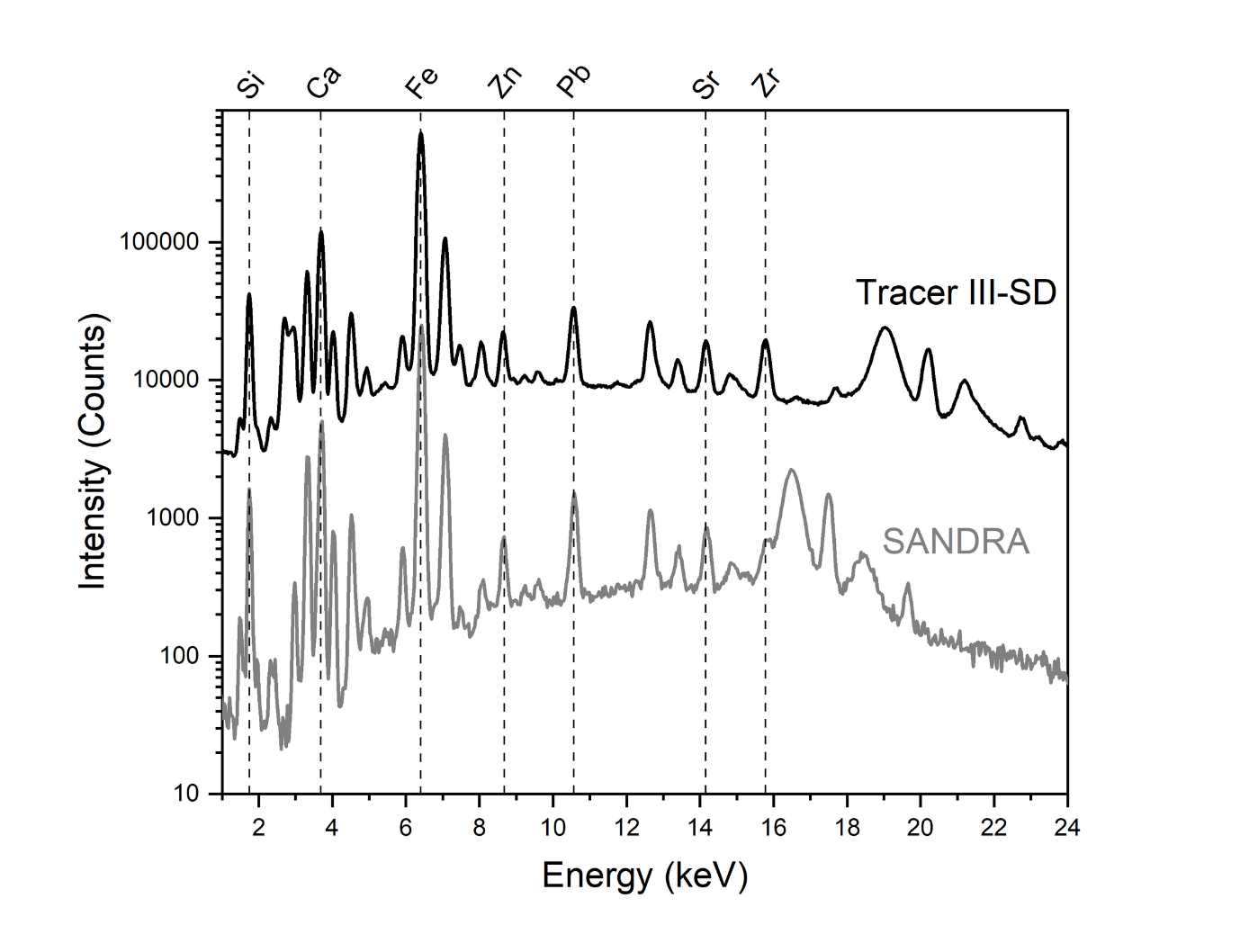
**

**Figure S3.** XRF spectra of the NIST SRM 2711 Montana soil. Comparison of detected elements under the experimental conditions described in the experimental section for SANDRA and Bruker Tracer III-SD X-ray spectrometers

**Table S1**. XRF elemental peak intensities (counts) of the *in situ* soil analyses at PLM-3. Intensities correspond to K lines (Al - Mn).

| # | **Al** | **Si** | **S** | **Cl** | **K** | **Ca** | **Ti** | **V** | **Cr** | **Mn** |
| --- | --- | --- | --- | --- | --- | --- | --- | --- | --- | --- |
| **1** | 3004 | 69296 | 56350 | 307312 | 127148 | 2.61E+06 | 149452 | 7282 | 4698 | 54398 |
| **2** | 1689 | 59511 | 37388 | 266446 | 134098 | 2.91E+06 | 131706 | 6408 | 2808 | 43155 |
| **3** | 3040 | 59428 | 50971 | 360971 | 143683 | 2.38E+06 | 174746 | 7868 | 3105 | 51734 |
| **4** | 2580 | 54423 | 24446 | 308940 | 85989 | 3.70E+06 | 109743 | 5339 | 1439 | 36234 |
| **5** | 1800 | 64583 | 51055 | 206884 | 136970 | 2.22E+06 | 184810 | 8879 | 3950 | 58056 |
| **6** | 3665 | 58562 | 66187 | 566460 | 124684 | 2.08E+06 | 208567 | 8616 | 2802 | 58056 |
| **7** | 2684 | 68509 | 34542 | 215815 | 118354 | 3.08E+06 | 118095 | 5368 | 3281 | 49429 |
| **8** | 3655 | 70659 | 21680 | 237690 | 131442 | 2.67E+06 | 133343 | 5944 | 2126 | 39281 |
| **10** | 1479 | 57347 | 22732 | 276172 | 94756 | 4.54E+06 | 75860 | 723 | 2803 | 31158 |
| **11** | 2217 | 42095 | 28100 | 323621 | 80919 | 3.41E+06 | 110645 | 4400 | 2046 | 37892 |
| **12** | 2467 | 63905 | 19390 | 250572 | 118697 | 3.12E+06 | 117211 | 5241 | 2662 | 36777 |
| **13** | 1866 | 52138 | 63156 | 432300 | 90409 | 2.24E+06 | 219967 | 11164 | 3523 | 61833 |
| **14** | 2416 | 49217 | 46713 | 305387 | 92360 | 2.09E+06 | 260710 | 13654 | 6874 | 69494 |
| **16** | 2574 | 52629 | 69600 | 322354 | 113433 | 1.94E+06 | 286013 | 12890 | 3654 | 65749 |
| **17** | 3206 | 74115 | 53957 | 430147 | 111655 | 1.88E+06 | 120415 | 5183 | 3258 | 45119 |
| **18** | 2234 | 71074 | 52394 | 280047 | 126615 | 2.85E+06 | 162324 | 6221 | 3716 | 47484 |
| **19** | 2508 | 63152 | 59506 | 222766 | 131476 | 2.74E+06 | 200055 | 8530 | 4849 | 59687 |
| **21** | 2883 | 73854 | 36787 | 237212 | 150941 | 3.20E+06 | 117595 | 6012 | 2736 | 37564 |
| **22** | 2888 | 65001 | 51451 | 351688 | 166589 | 2.42E+06 | 179575 | 8127 | 2468 | 42000 |

**Table S2**. XRF elemental peak intensities (counts) of the *in situ* soil analyses at PLM-3. Intensities correspond to K lines (Fe - Zr).

| # | **Fe** | **Ni** | **Cu** | **Zn** | **As** | **Rb** | **Sr** | **Y** | **Zr** |
| --- | --- | --- | --- | --- | --- | --- | --- | --- | --- |
| **1** | 3.34E+06 | 50022 | 41188 | 21878 | 7437 | 14703 | 297007 | 5007 | 71582 |
| **2** | 3.16E+06 | 48142 | 40206 | 20496 | 7150 | 15989 | 326385 | 4640 | 105142 |
| **3** | 3.72E+06 | 52104 | 41959 | 19632 | 7922 | 16121 | 279836 | 4678 | 58513 |
| **4** | 2.41E+06 | 48510 | 40344 | 16470 | 6490 | 12826 | 328279 | 7582 | 74328 |
| **5** | 4.08E+06 | 47096 | 37340 | 21559 | 8634 | 13913 | 259928 | 5778 | 81805 |
| **6** | 3.83E+06 | 52758 | 46233 | 21992 | 9097 | 12376 | 233555 | 5799 | 83639 |
| **7** | 2.93E+06 | 51476 | 41083 | 20258 | 7625 | 16143 | 327212 | 4287 | 54242 |
| **8** | 2.78E+06 | 50830 | 42897 | 22757 | 9651 | 19230 | 286533 | 4662 | 84246 |
| **10** | 1.81E+06 | 42805 | 40019 | 18496 | 6160 | 14222 | 370460 | 3215 | 46336 |
| **11** | 2.44E+06 | 46069 | 40281 | 19120 | 3361 | 12002 | 313134 | 3434 | 65510 |
| **12** | 2.65E+06 | 47827 | 43453 | 21647 | 5171 | 15555 | 297792 | 4147 | 80312 |
| **13** | 4.15E+06 | 48069 | 42007 | 19549 | 8107 | 9149 | 241387 | 5392 | 107156 |
| **14** | 5.14E+06 | 44141 | 36854 | 21084 | 7785 | 8195 | 228469 | 4560 | 86047 |
| **16** | 4.76E+06 | 45982 | 36538 | 19799 | 7620 | 9753 | 224879 | 4529 | 142117 |
| **17** | 2.97E+06 | 53733 | 47594 | 24846 | 10328 | 18161 | 270111 | 5205 | 85270 |
| **18** | 3.35E+06 | 51431 | 41766 | 20869 | 8345 | 13355 | 287507 | 5138 | 113480 |
| **19** | 3.81E+06 | 48719 | 38671 | 20615 | 8561 | 12192 | 269058 | 4534 | 97878 |
| **21** | 2.64E+06 | 49824 | 41932 | 18137 | 8850 | 18317 | 320173 | 4062 | 46992 |
| **22** | 3.18E+06 | 51245 | 45089 | 20576 | 8100 | 17516 | 276218 | 3382 | 62272 |

***Suppl. 3. FTIR measurements***

The FTIR spectra of all the samples analyzed, both unwashed and washed (Figures S4-S7) exhibit a similar spectral profile dominated by characteristic proteinaceous bands (amide I and II, NH, OH, CH, CS).

The following vibrational analysis is performed using the spectrum of the unwashed sample 799 (Figure S4a). In particular, the signal around 3274 cm^-1^ is attributed to the combination of the υNH and υOH vibrational modes. This signal has been reported by Otłowska et al. [[1]](https://www.zotero.org/google-docs/?caQoST), who associate the υNH vibration with an amino acid residue, while the υOH vibration is associated with a phenolic ring. The bands at 2927 and 2859 cm^-1^ are attributed to the asymmetric stretching of CH fragments associated with aliphatic chains in the protein. Two characteristic intense bands centered around 1629 and 1522 cm^-1^ are attributed to amide I and II vibrations, respectively. These bands have been reported as vibrations related to the secondary structure of the keratin protein, typical of animal fibers [[1–4]](https://www.zotero.org/google-docs/?OUrwtJ).

In particular, the spectral overlap around the bands at 1040, 1229, 1400 cm^-1^, among others, prevents a precise assignment of bands related to the dyes. Vibrational signals at 1443 and 1400 cm^-1^ are assigned to the deformation CH_2_ and CH_3_ fragments, respectively. The 1229 cm^-1^ band is attributed to C-O-C stretching in proteins, lipids, or detergents [[2]](https://www.zotero.org/google-docs/?pMnta1). The bands at 1040 cm^-1^ and 1074 cm^-1^ have been described by Rajkowska et al. [[4]](https://www.zotero.org/google-docs/?ZMBFIP) as vibrational signals associated with cysteine residues. This indicates that both bands are a product of the oxidation of the disulfide bridge of the cysteine residue, which is transformed into cysteic acid (-SO-) and cystine monoxide (-SO-S-), respectively. Finally, the vibrational signal at 521 cm^-1^ is ascribed to a -C-S- stretching mode.

Samples 804, 806, and 807's vibrational behavior is similar to that described in the previous paragraphs (Figures S5-S7).


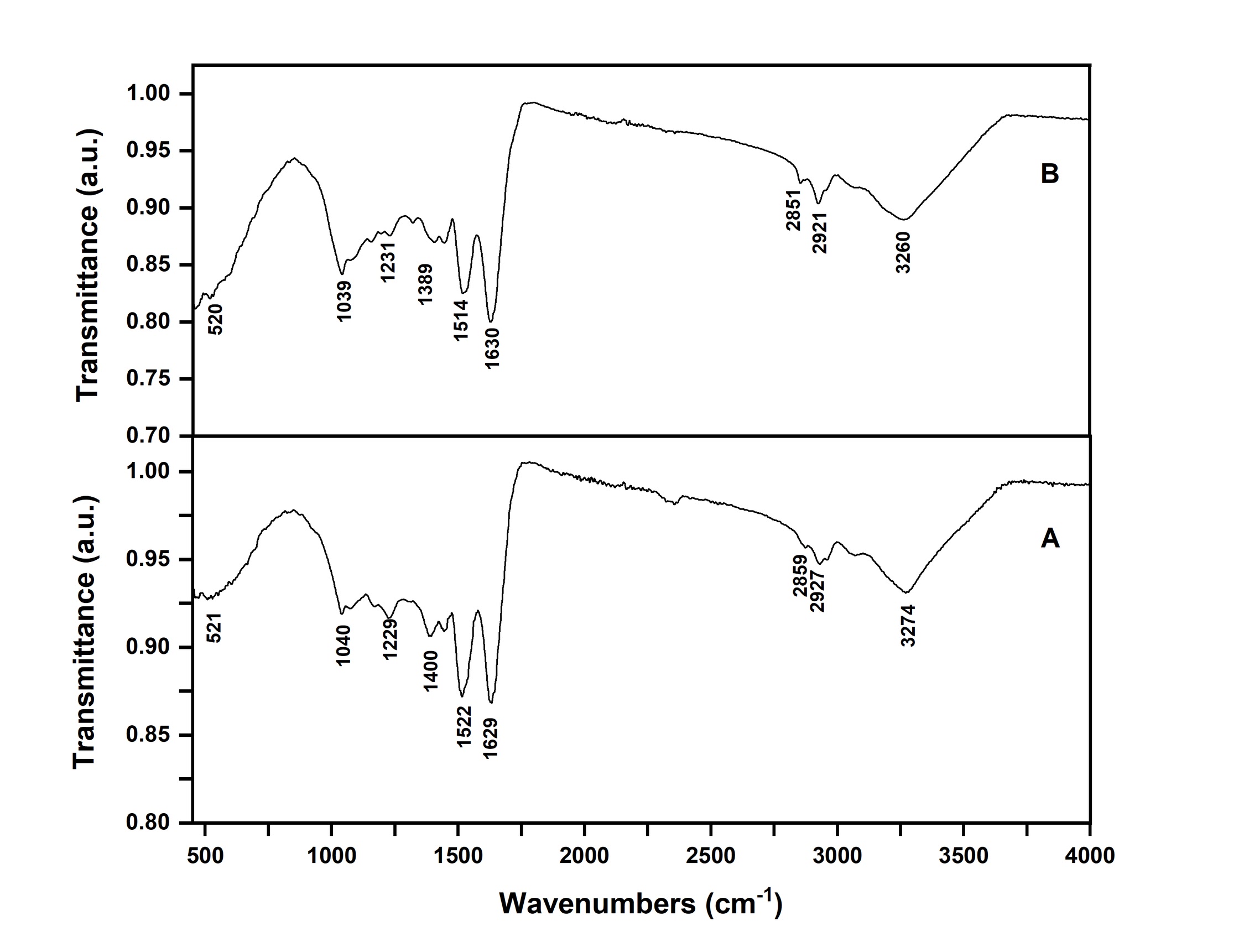


**Figure S4.** FTIR-ATR spectra of sample 799, (A) unwashed and (B) washed


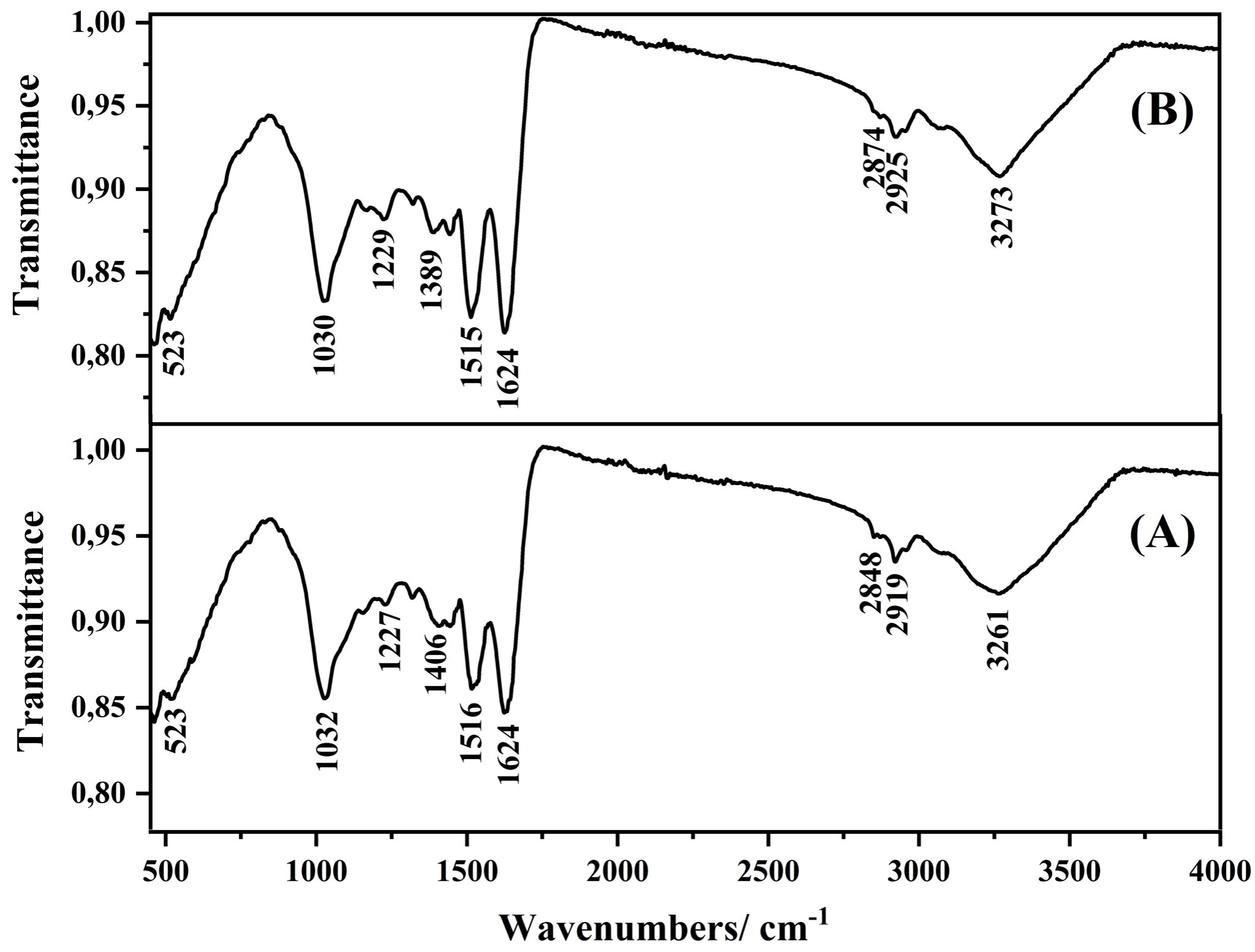


**Figure S5.** FTIR-ATR spectra of sample 804, (A) unwashed and (B) washed


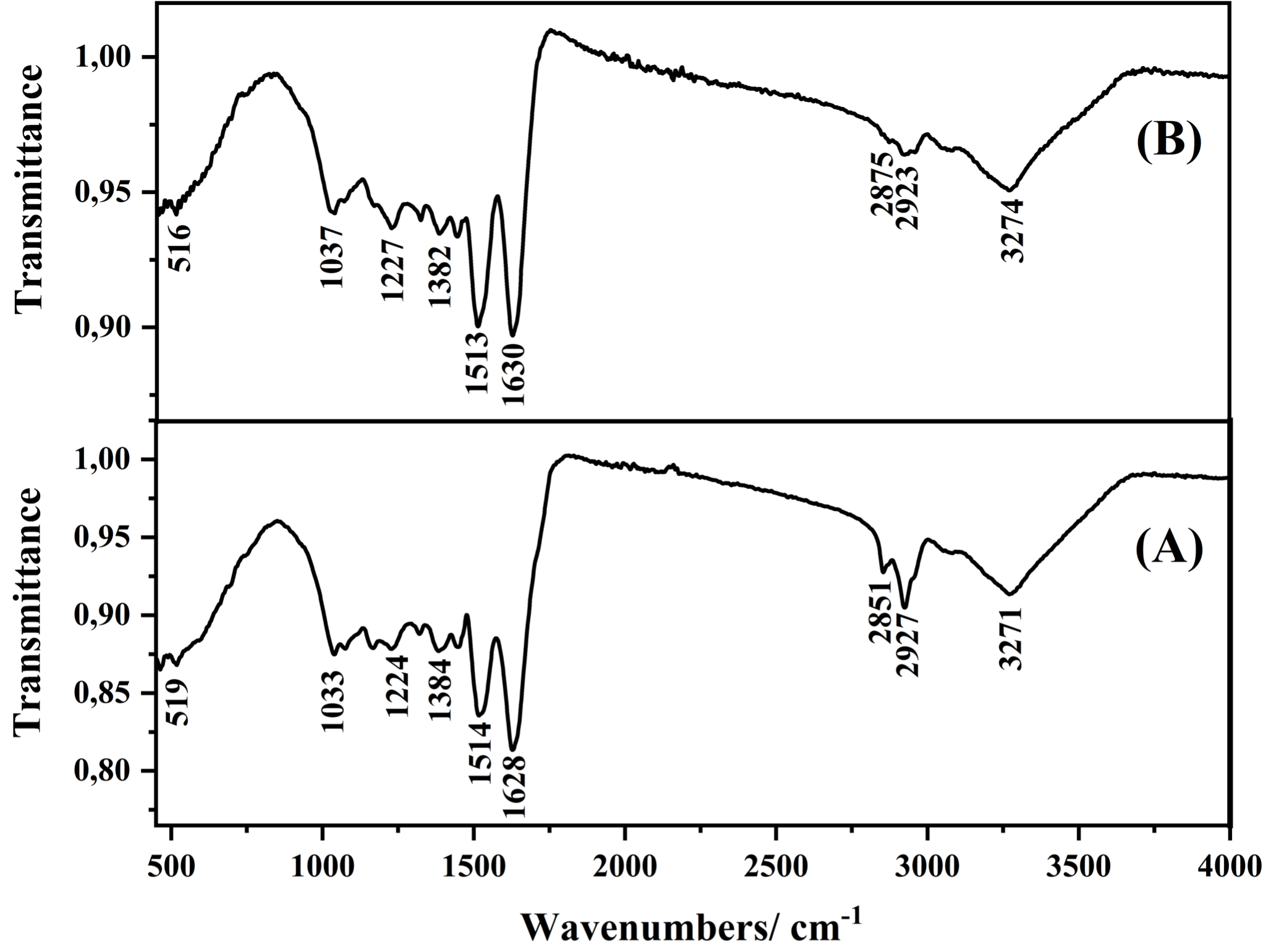


**Figure S6.** FTIR-ATR spectra of sample 806, (A) unwashed and (B) washed


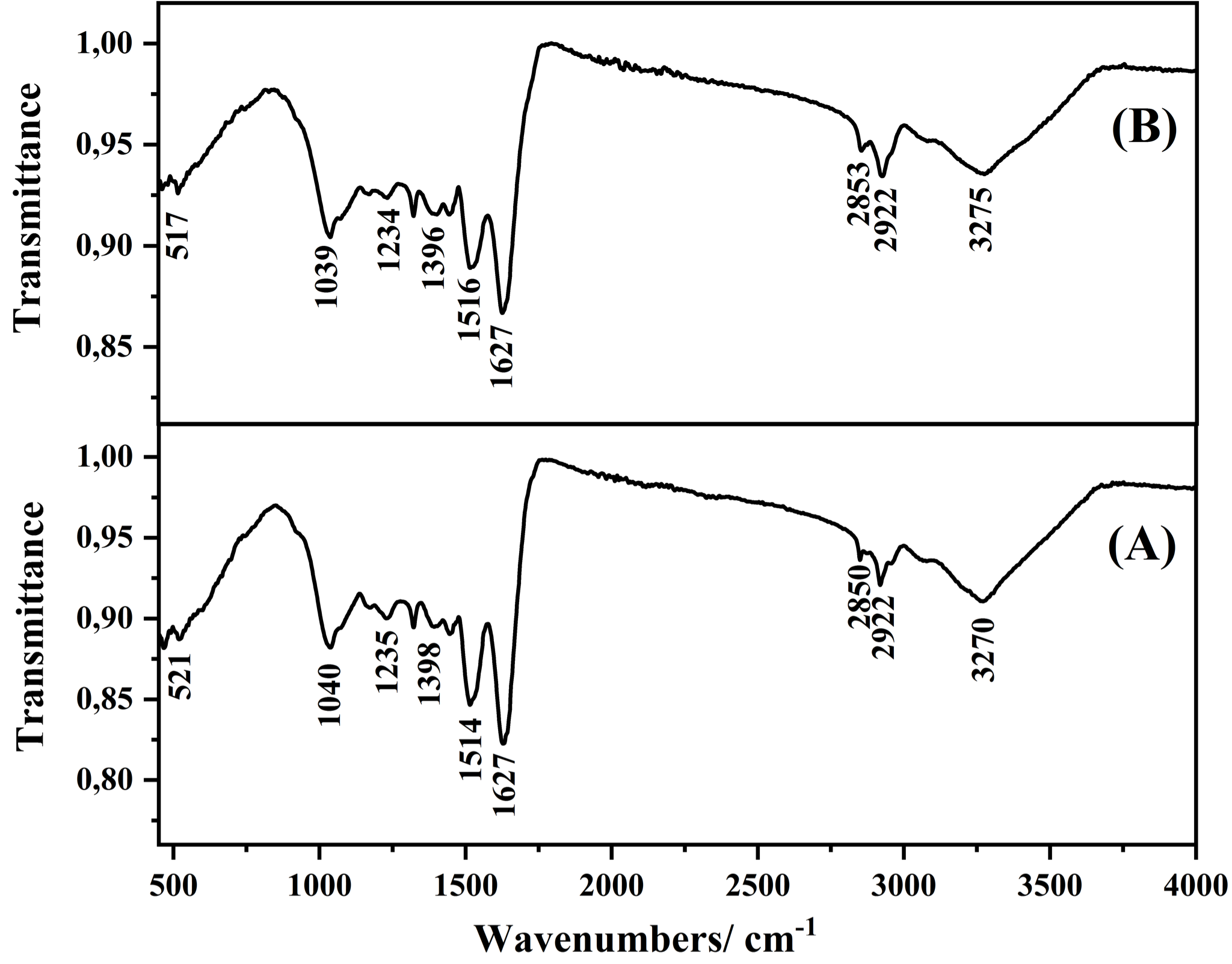


**Figure S7.** FTIR-ATR spectra of sample 807, (A) unwashed and (B) washed

***Suppl. 4. SERS measurements***


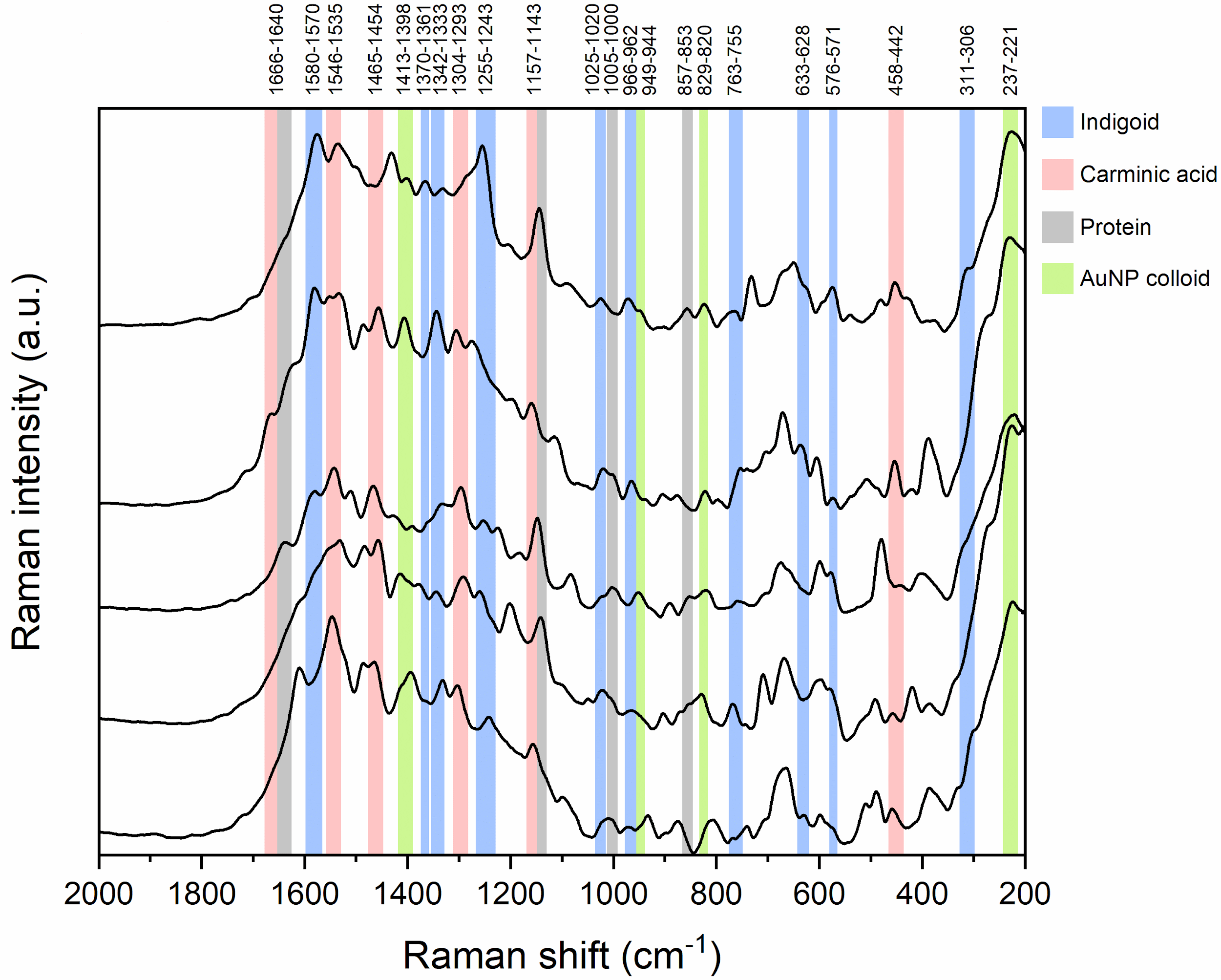


**Figure S8**: SERS spectra of fibre 799


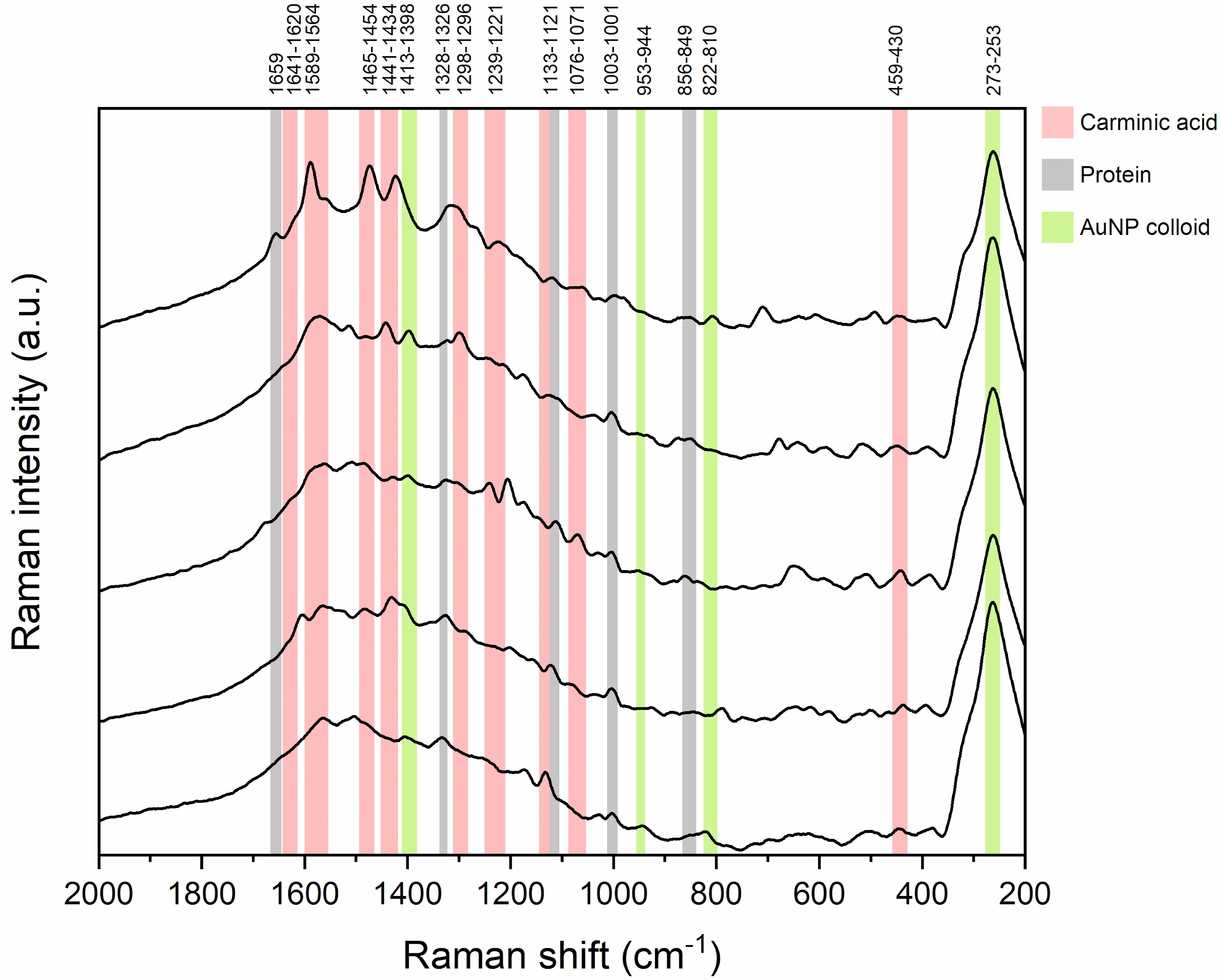


**Figure S9**: SERS spectra of fibre 804


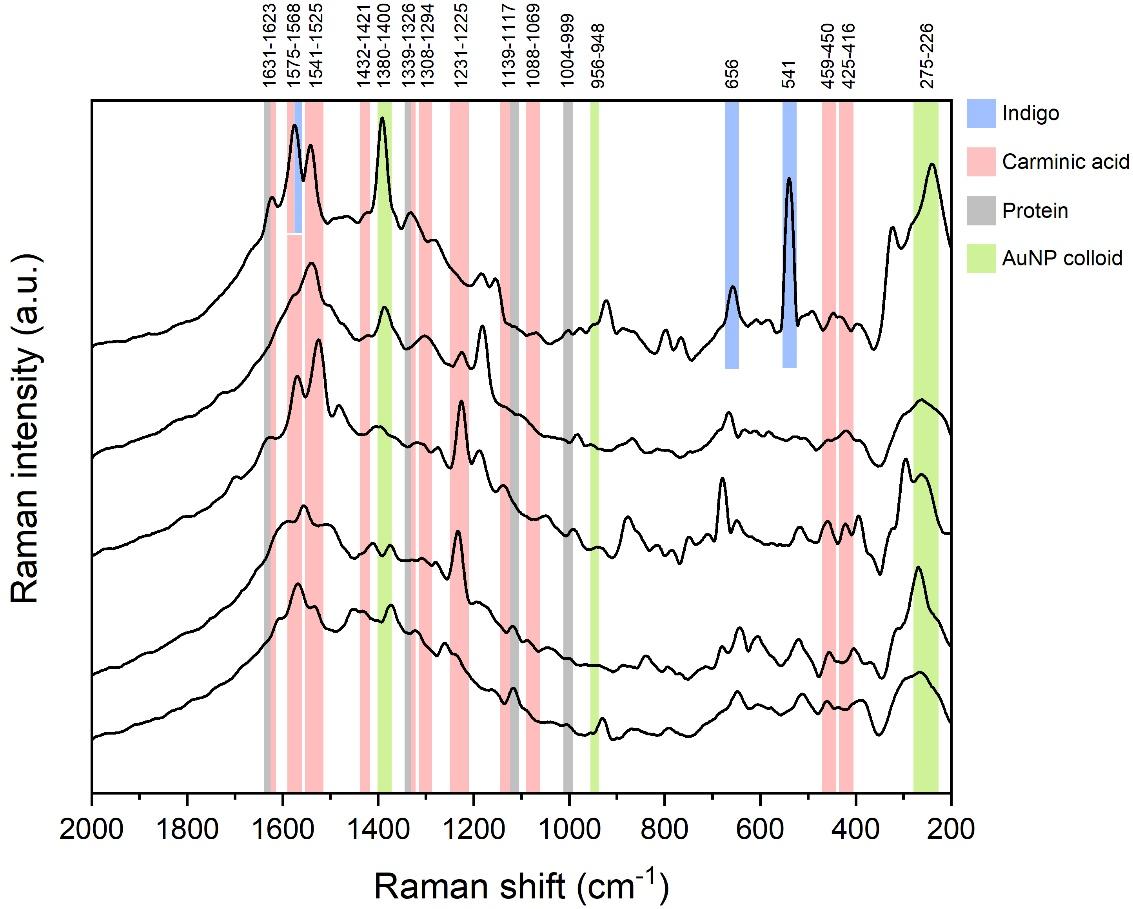


**Figure S10**: SERS spectra of fibre 806


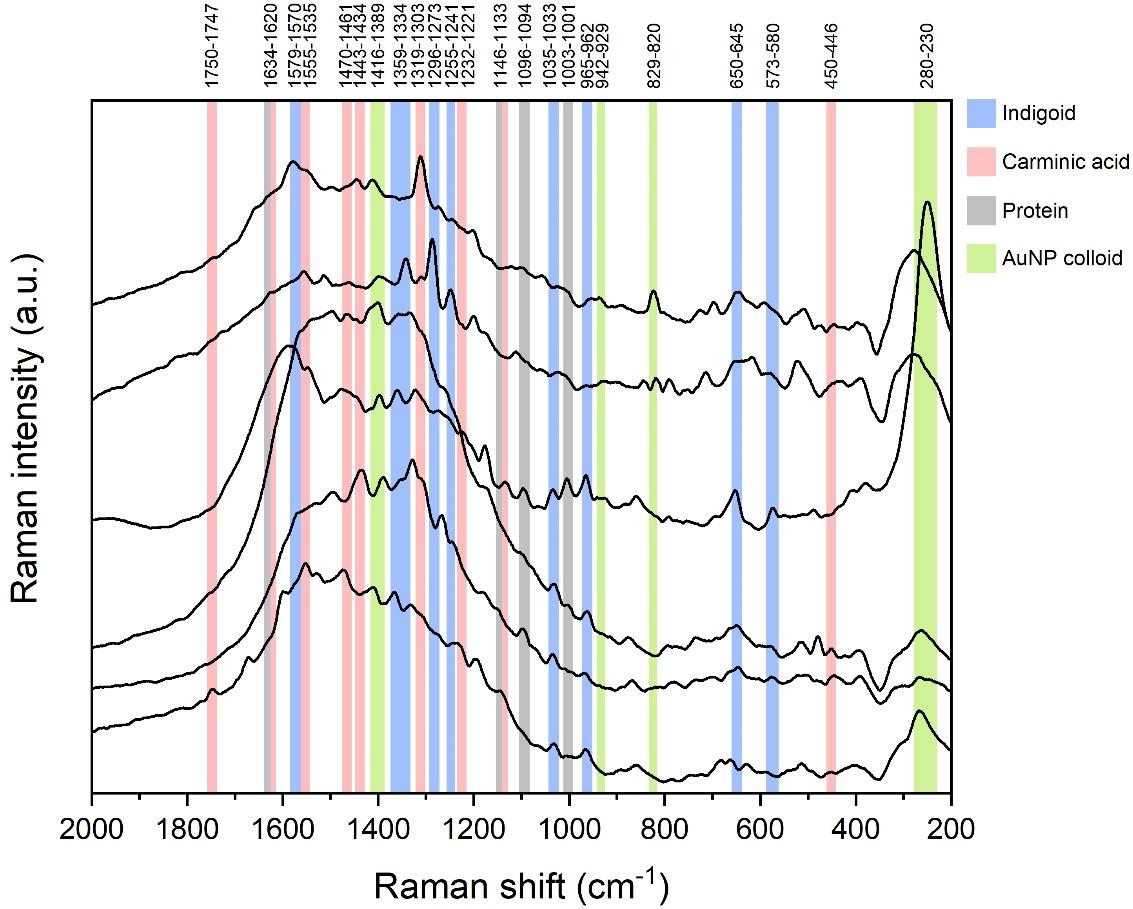


**Figure S11**: SERS spectra of fibre 807


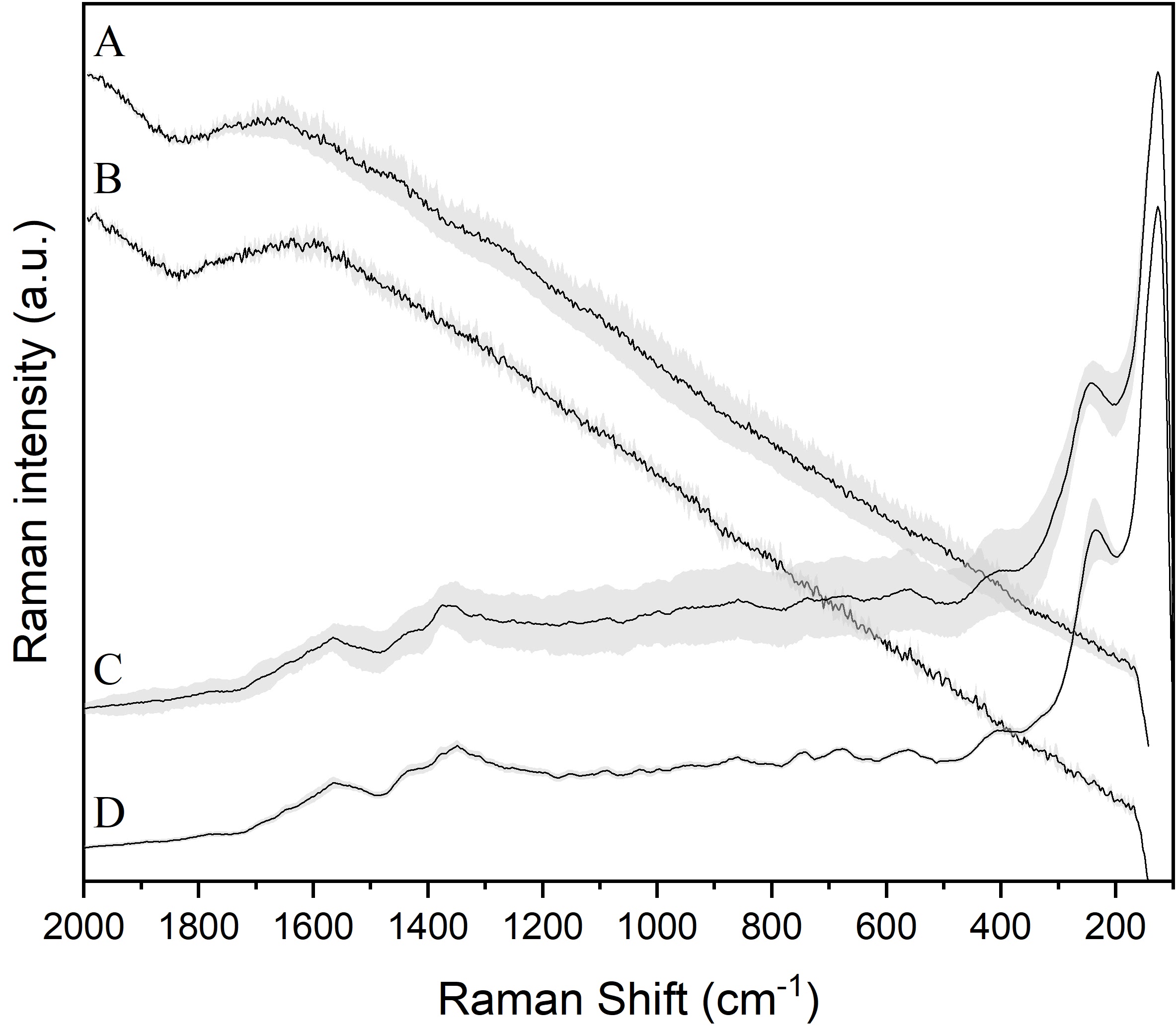


**Figure S12**: 532 nm SERS spectra of an undyed wool fibre using gold nanoparticles (A) and the gold colloid (B); 785 nm SERS spectra of an undyed wool fibre using gold nanoparticles (C) and the gold colloid (D)

***Suppl. 5. Modern textile analysis***


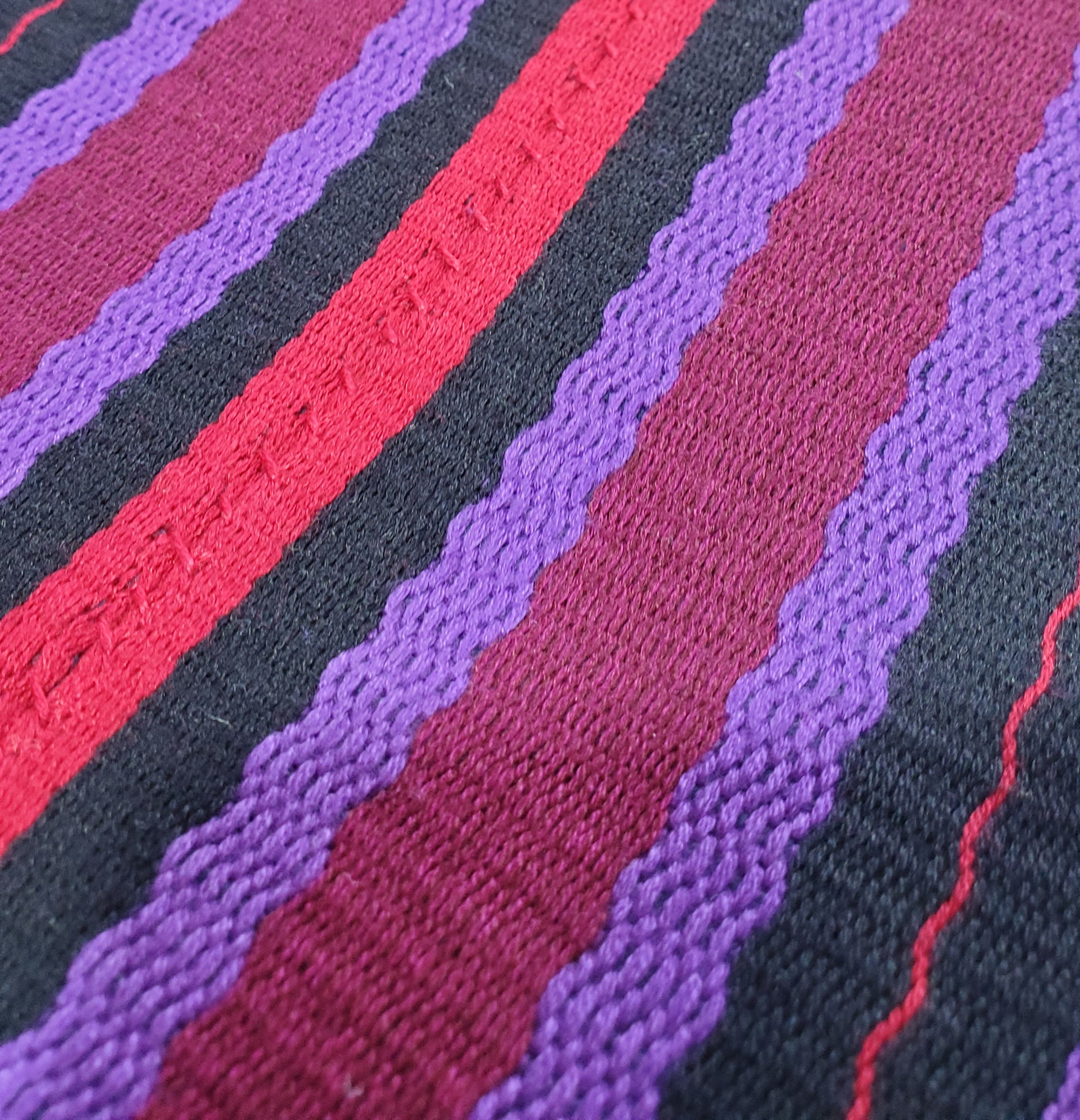


**Figure S13**: Modern textile dyed with shellfish purple (purple areas)


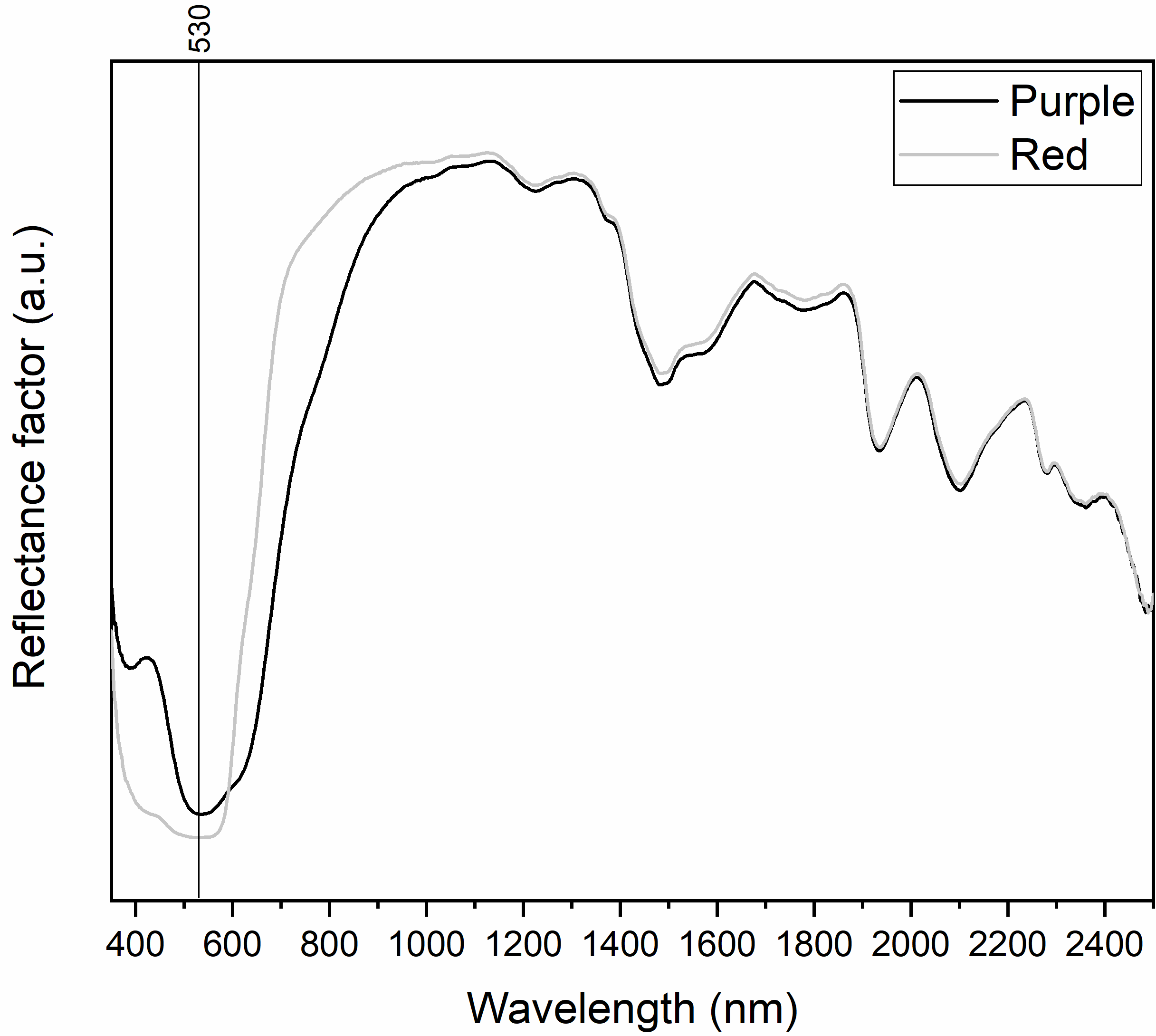


**Figure S14**: FORS spectra from the red and purple areas of a modern cotton textile. Purple areas were dyed with shellfish purple


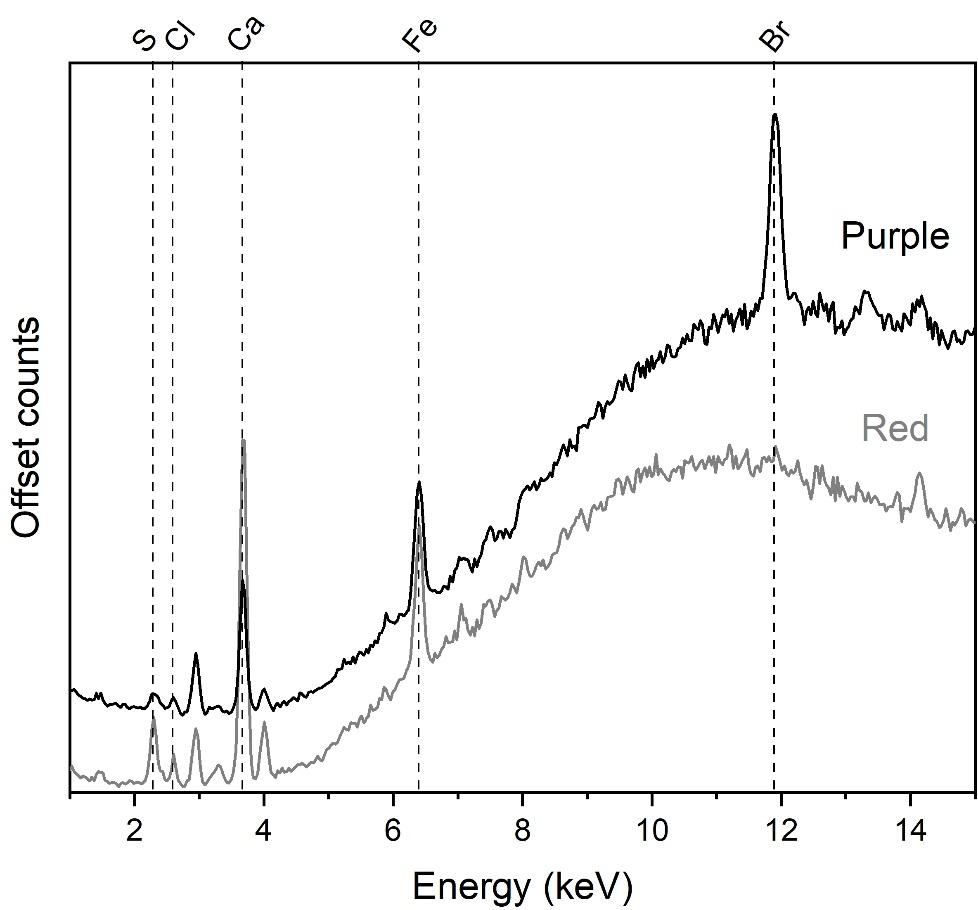


**Figure S15**: XRF spectra from the red and purple areas of a modern cotton textile. Purple areas were dyed with shellfish purple

**REFERENCES**

[1. Otłowska O, Ślebioda M, Kot-Wasik A, Karczewski J, Śliwka-Kaszyńska M. Chromatographic and Spectroscopic Identification and Recognition of Natural Dyes, Uncommon Dyestuff Components, and Mordants: Case Study of a 16th Century Carpet with Chintamani Motifs. Molecules. 2018;23: 339. doi:10.3390/molecules23020339](https://www.zotero.org/google-docs/?D4UyRL)

[2. Bertrand L, Vichi A, Doucet J, Walter P, Blanchard P. The fate of archaeological keratin fibres in a temperate burial context: microtaphonomy study of hairs from Marie de Bretagne (15th c., Orléans, France). J Archaeol Sci. 2014;42: 487–499. doi:10.1016/j.jas.2013.11.028](https://www.zotero.org/google-docs/?D4UyRL)

[3. Wojciechowska E, Włochowicz A, Wesełucha-Birczyńska A. Application of Fourier-transform infrared and Raman spectroscopy to study degradation of the wool fiber keratin. J Mol Struct. 1999;511–512: 307–318. doi:10.1016/S0022-2860(99)00173-8](https://www.zotero.org/google-docs/?D4UyRL)

[4. Rajkowska K, Otlewska A, Guiamet PS, Wrzosek H, Machnowski W. Pre-Columbian Archeological Textiles: A Source of Pseudomonas aeruginosa with Virulence Attributes. Appl Sci. 2019;10: 116. doi:10.3390/app10010116](https://www.zotero.org/google-docs/?D4UyRL)
