# Supplementary figures and images for: New insights into archaeological textiles (1000–1450AD) from the coastal region of the Atacama Desert: Preliminary evidence of a cochineal and shellfish purple dye combination
Source: PLoS One. 2025 Jun 4;20(6):e0325623. doi: 10.1371/journal.pone.0325623 (PMC12136422; doi:10.1371/journal.pone.0325623)

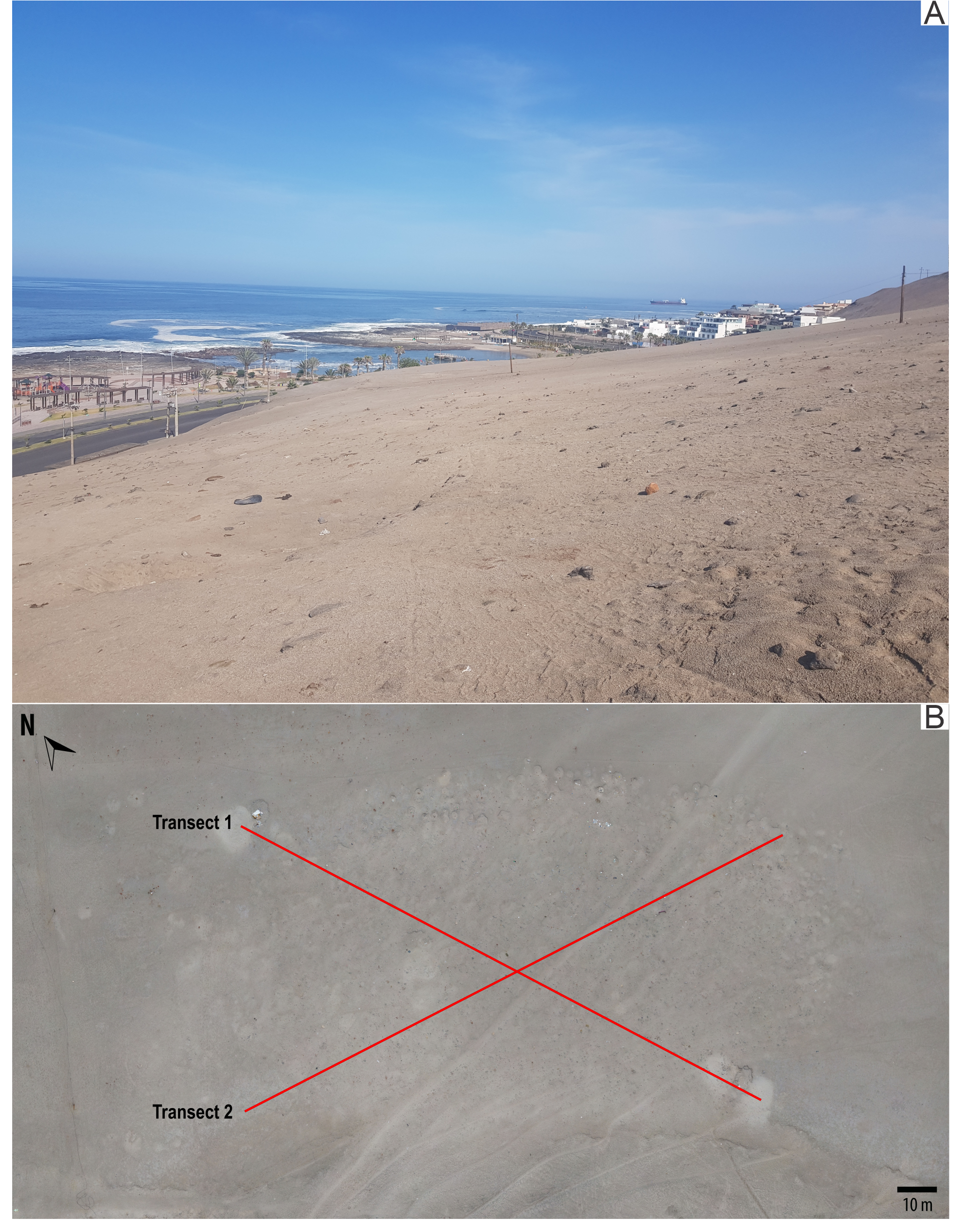

Supplement: S1 Fig — (TIF) [file pone.0325623.s002.tif]

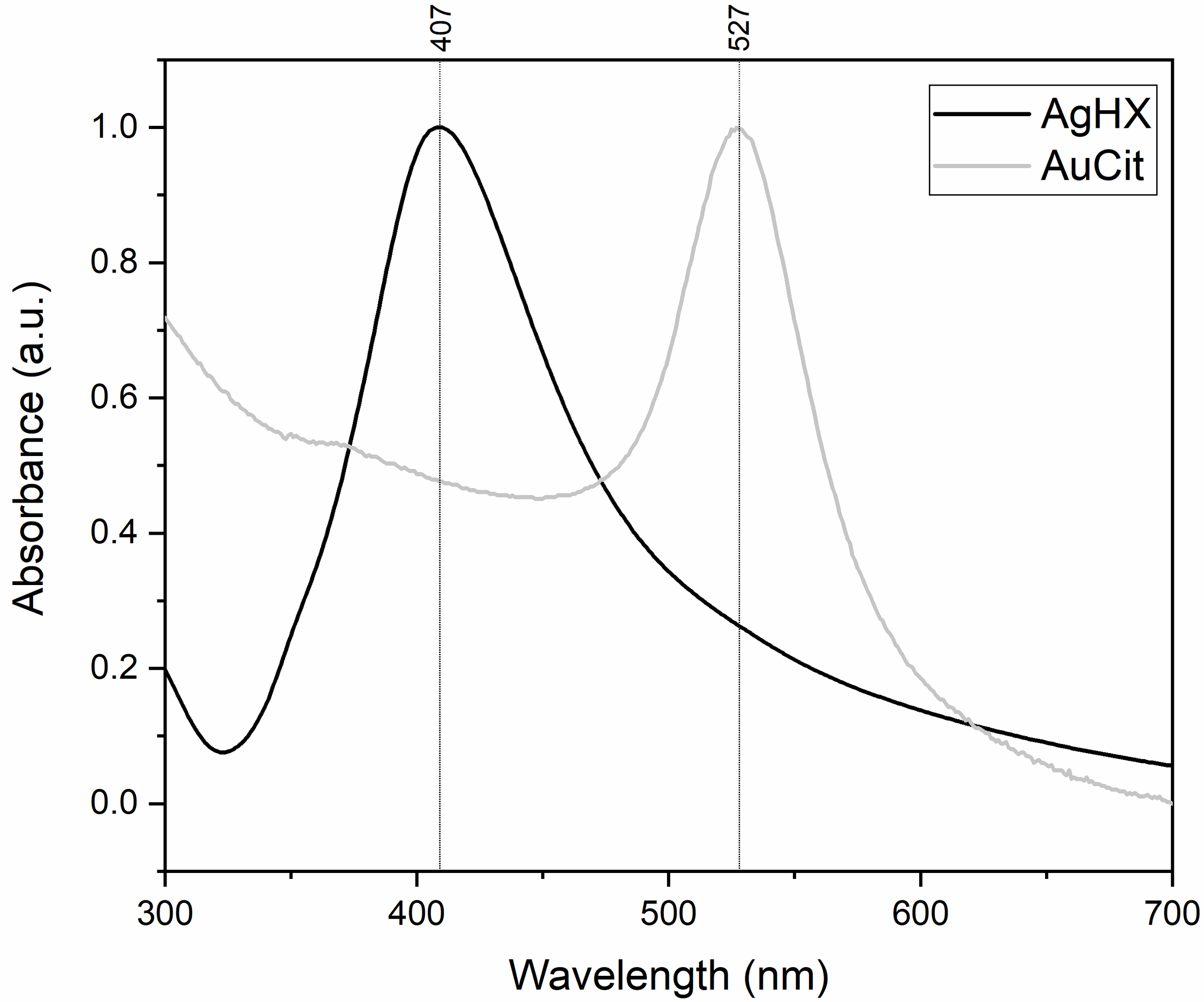

Supplement: S2 Fig — (TIF) [file pone.0325623.s003.tif]

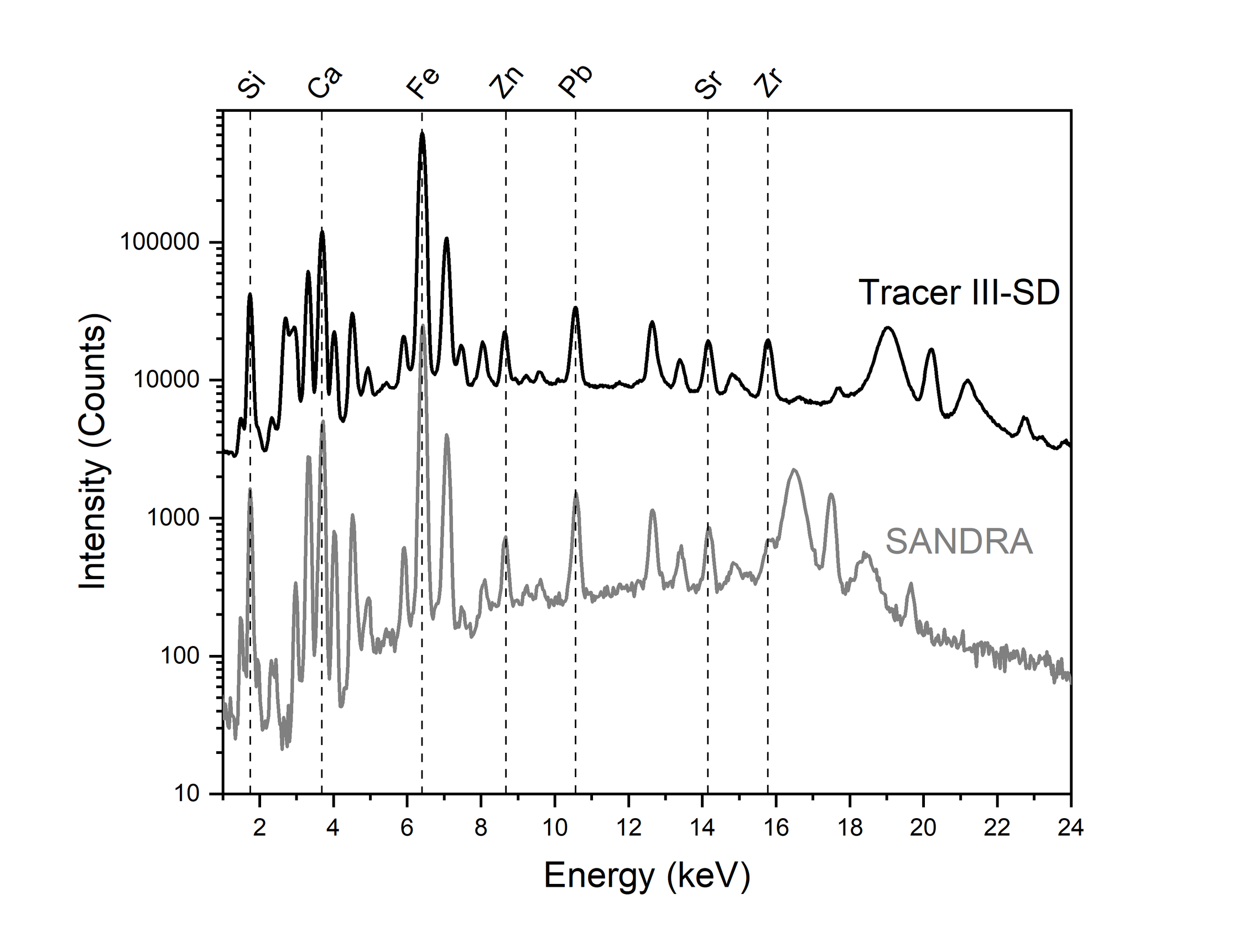

Supplement: S3 Fig — Comparison of detected elements under the experimental conditions described in the experimental section for SANDRA and Bruker Tracer III-SD X-ray spectrometers. (TIF) [file pone.0325623.s004.tif]

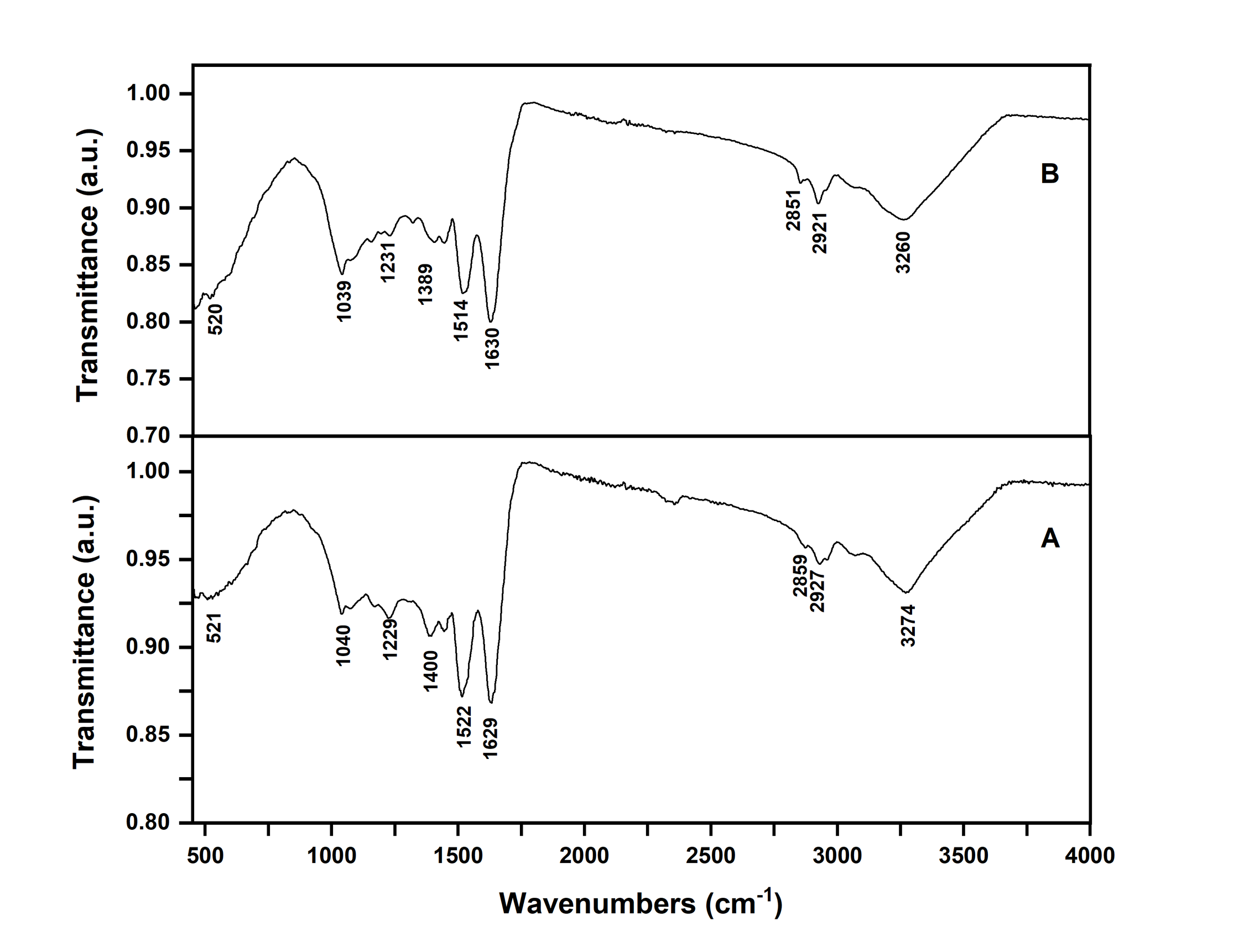

Supplement: S4 Fig — (TIF) [file pone.0325623.s005.tif]

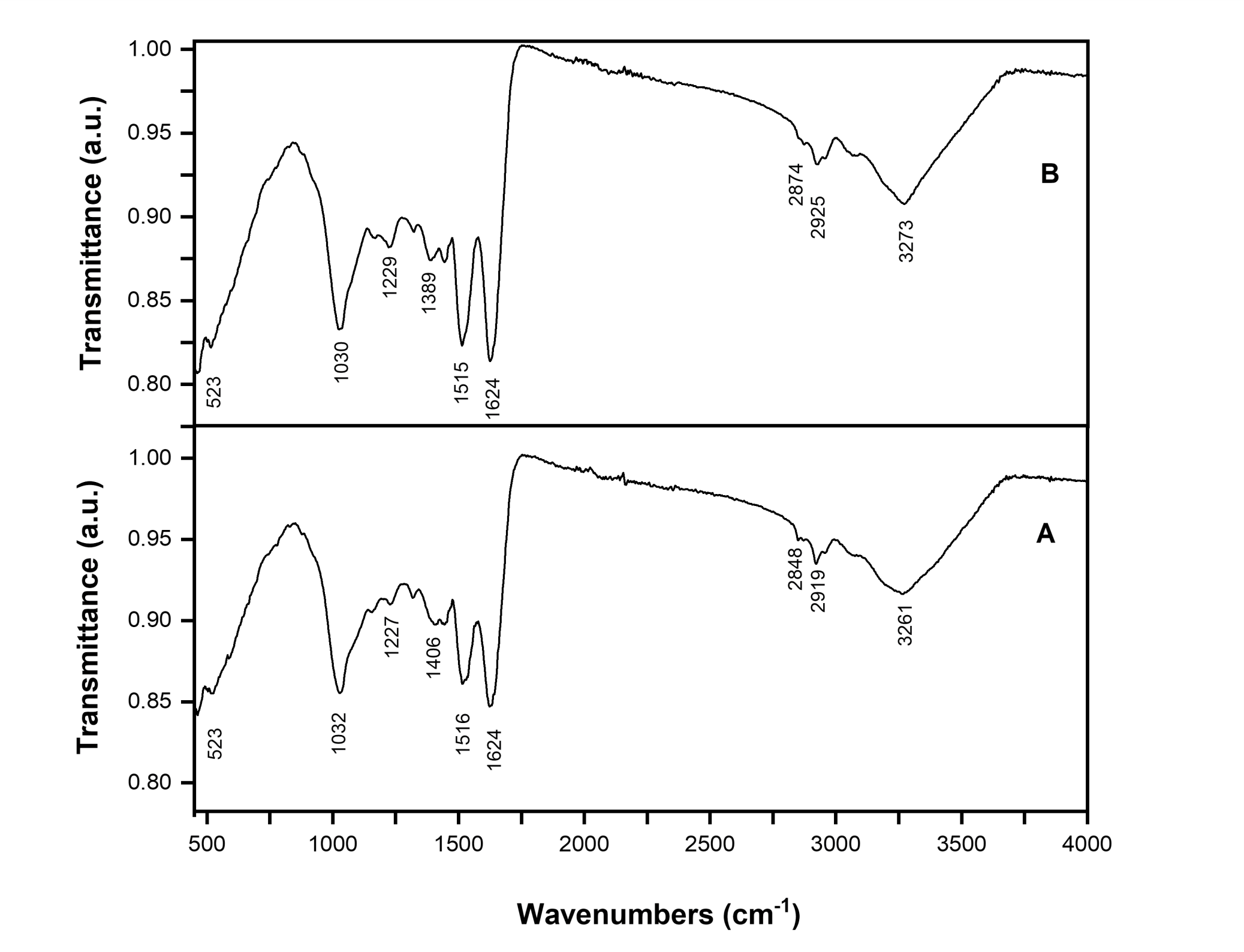

Supplement: S5 Fig — (TIF) [file pone.0325623.s006.tif]

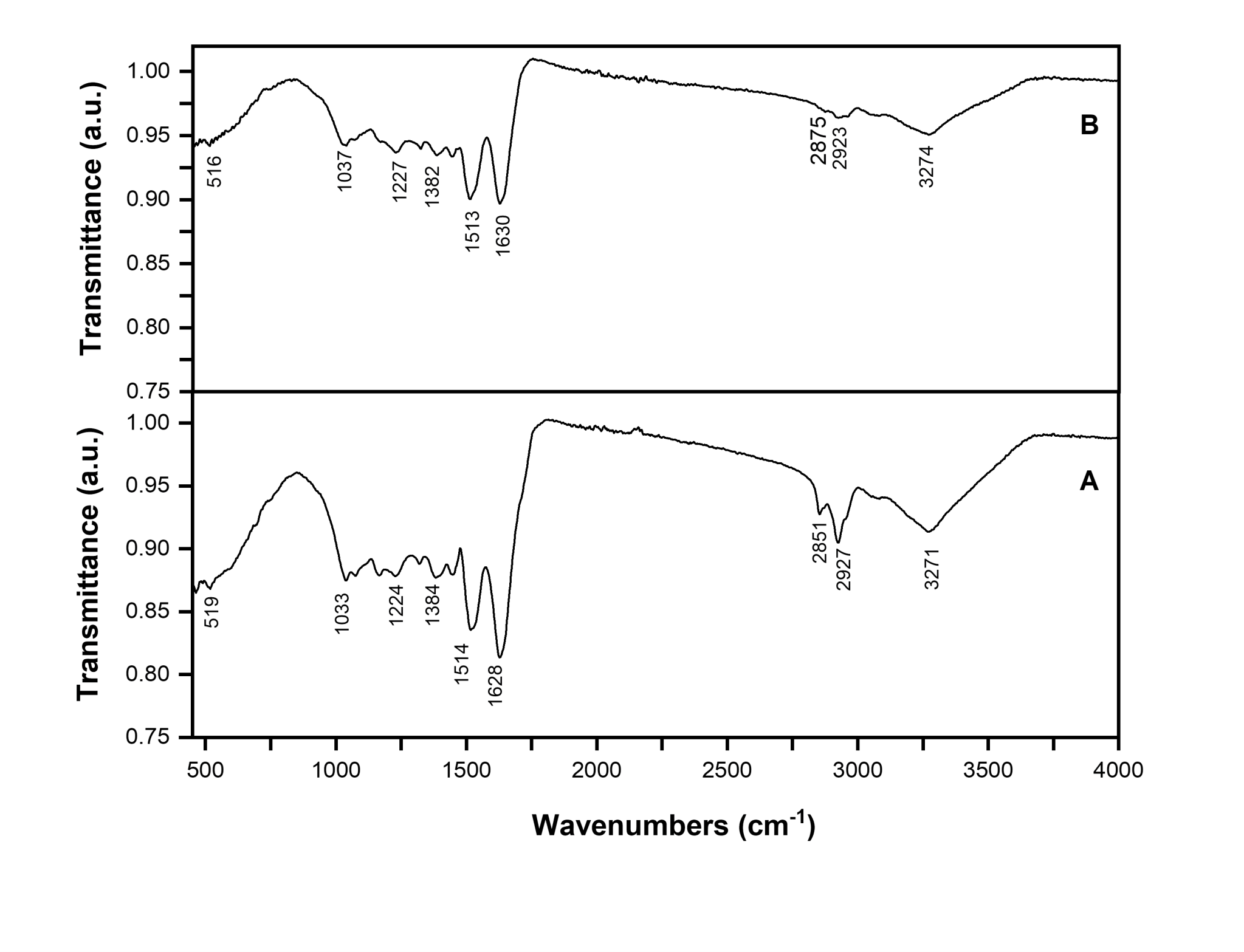

Supplement: S6 Fig — (TIF) [file pone.0325623.s007.tif]

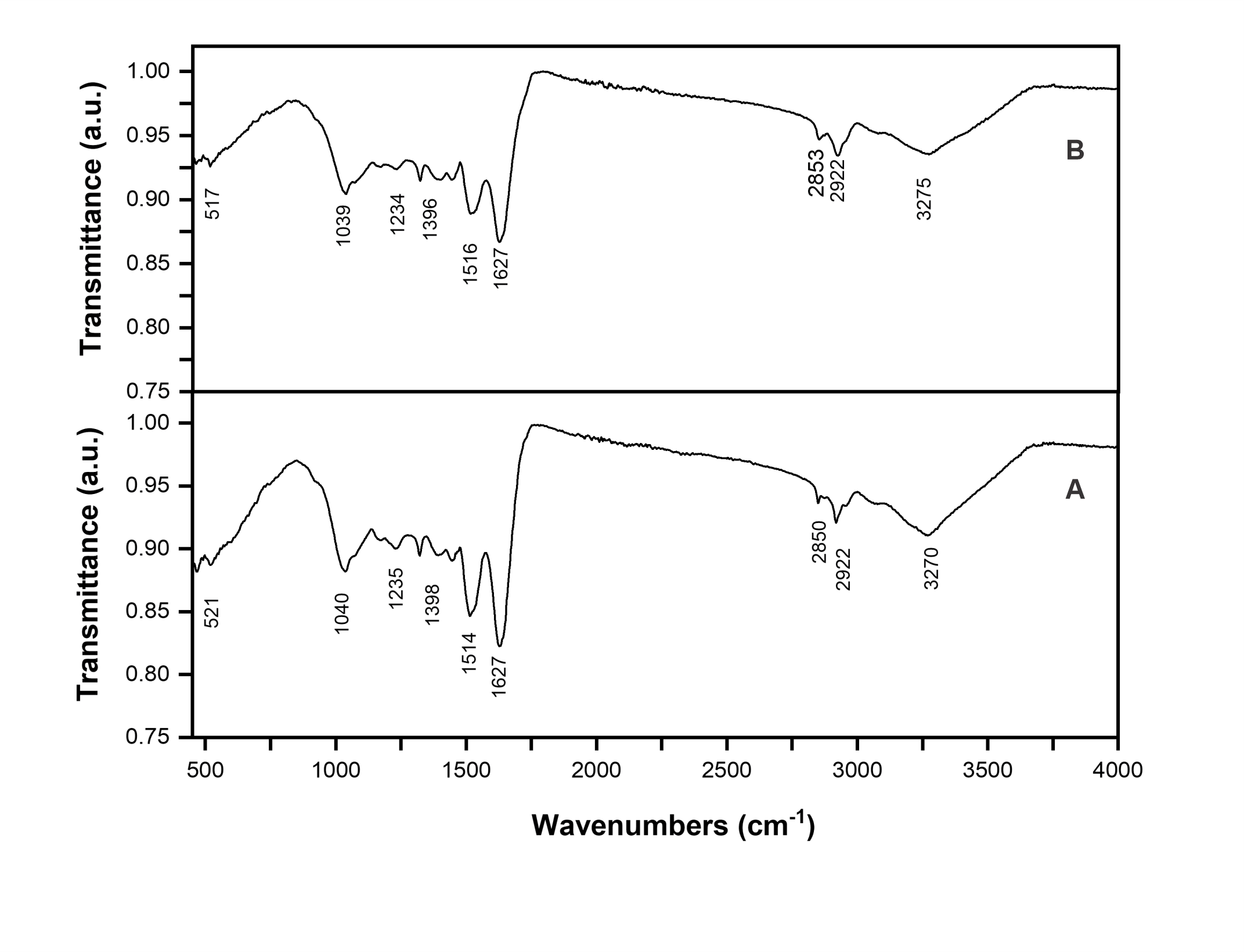

Supplement: S7 Fig — (TIF) [file pone.0325623.s008.tif]

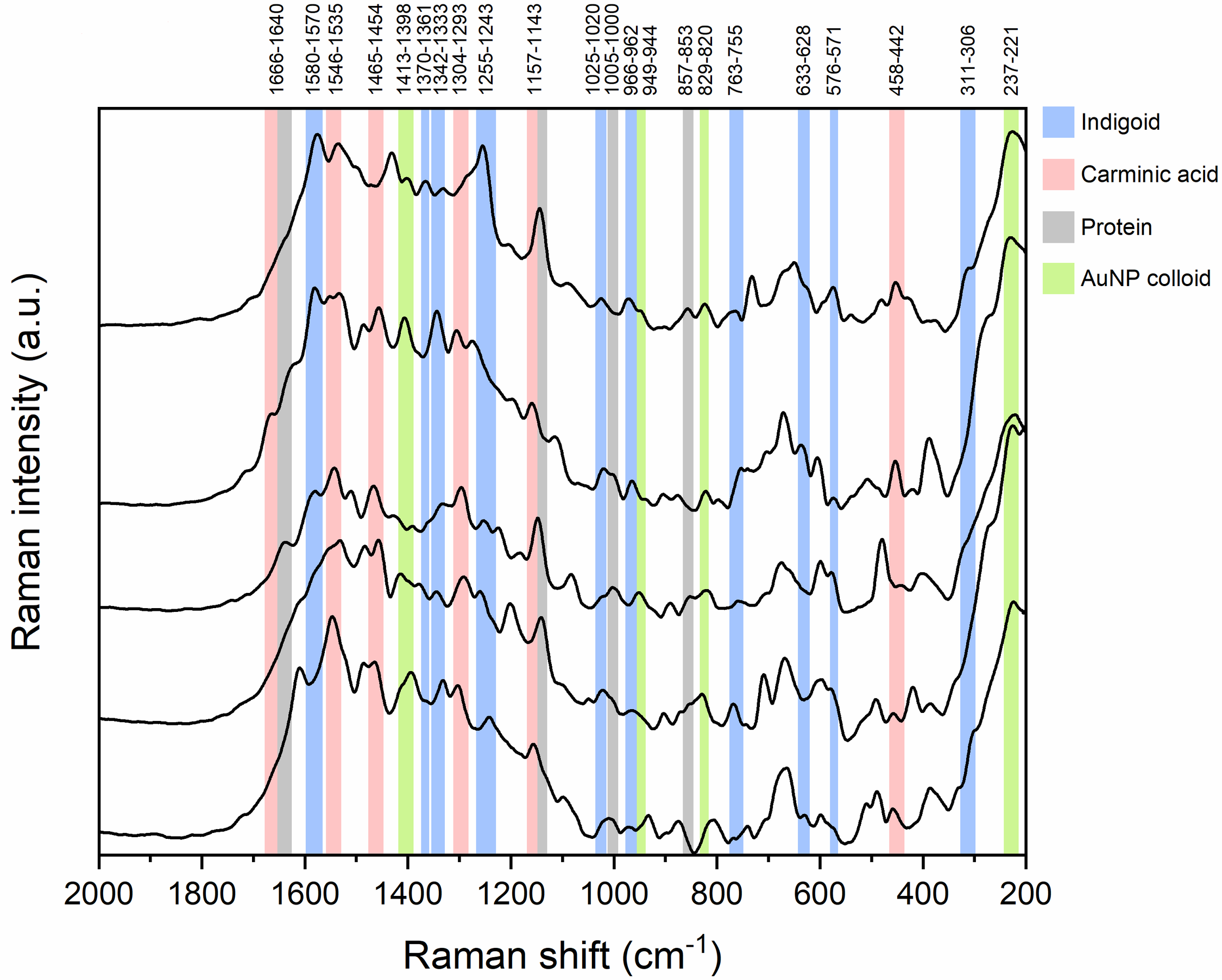

Supplement: S8 Fig — (TIF) [file pone.0325623.s009.tif]

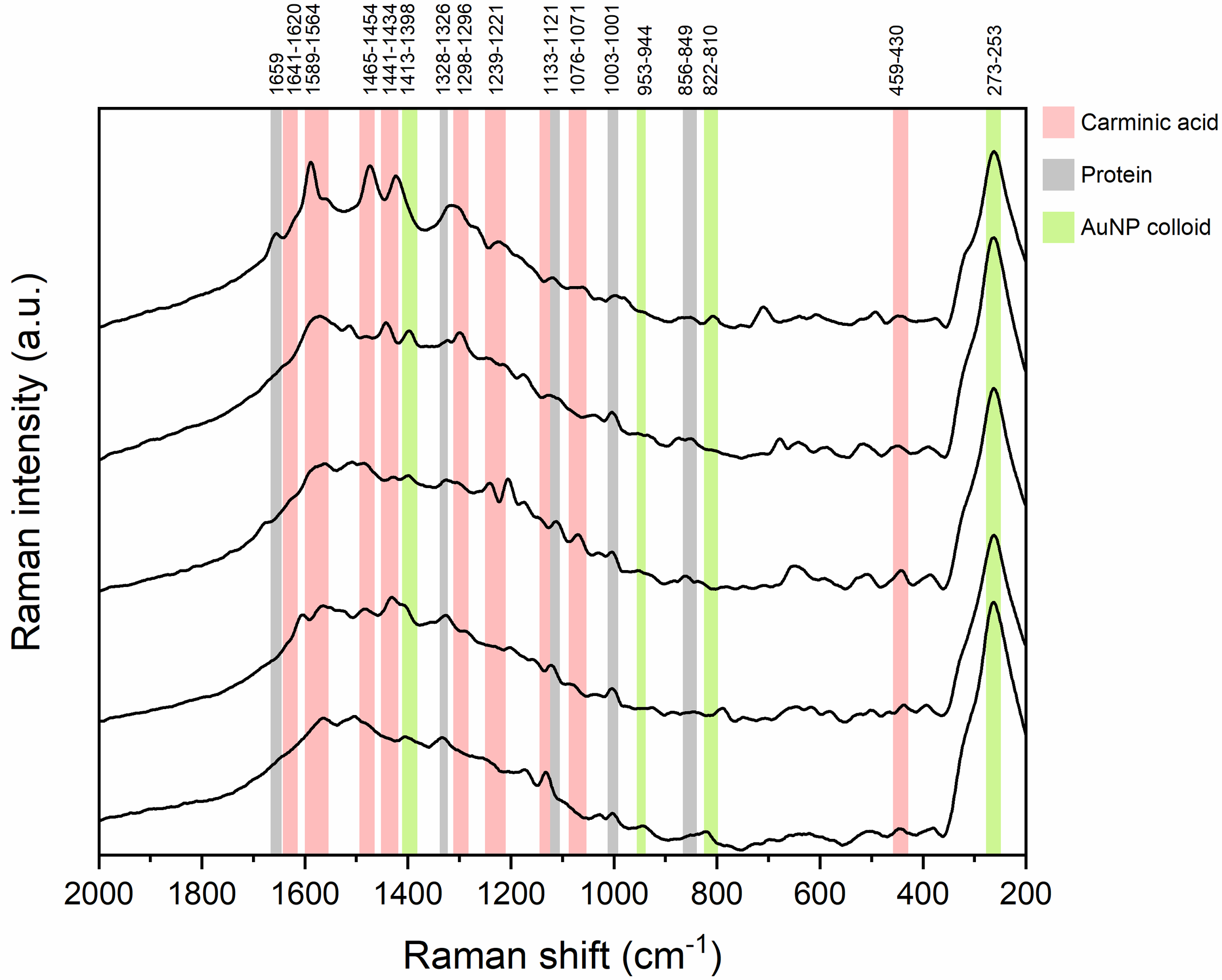

Supplement: S9 Fig — (TIF) [file pone.0325623.s010.tif]

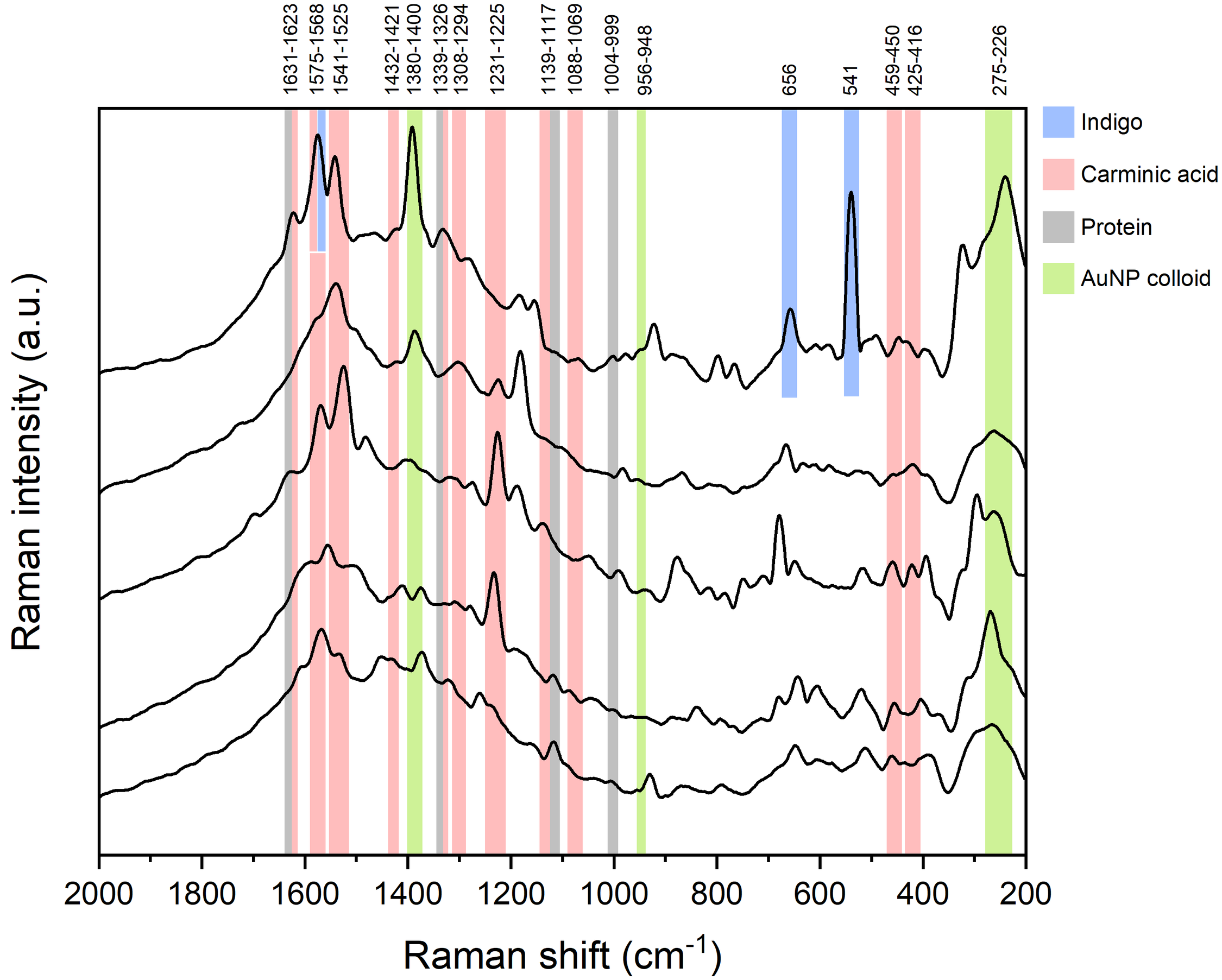

Supplement: S10 Fig — (TIF) [file pone.0325623.s011.tif]

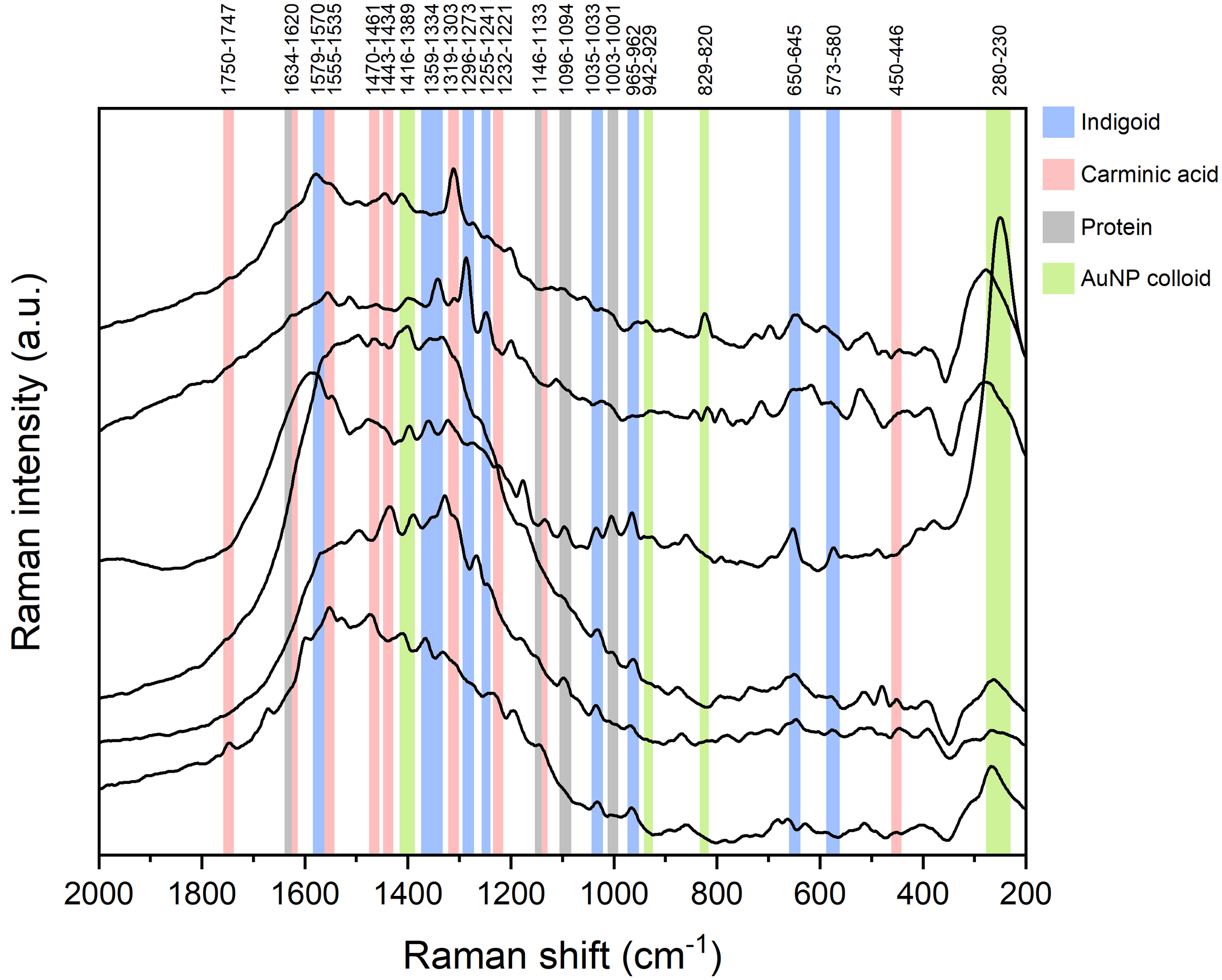

Supplement: S11 Fig — (TIF) [file pone.0325623.s012.tif]

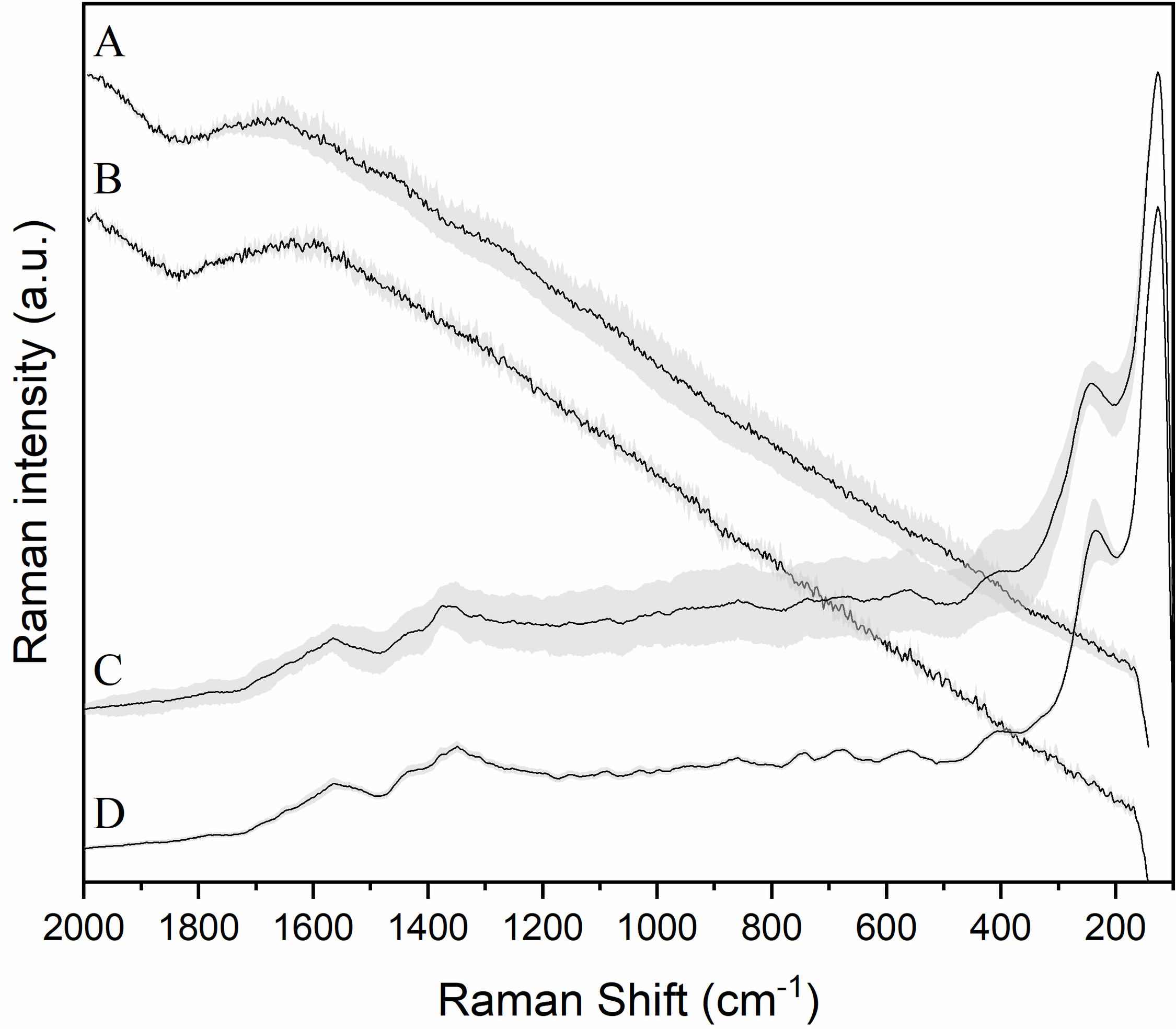

Supplement: S12 Fig — (TIF) [file pone.0325623.s013.tif]

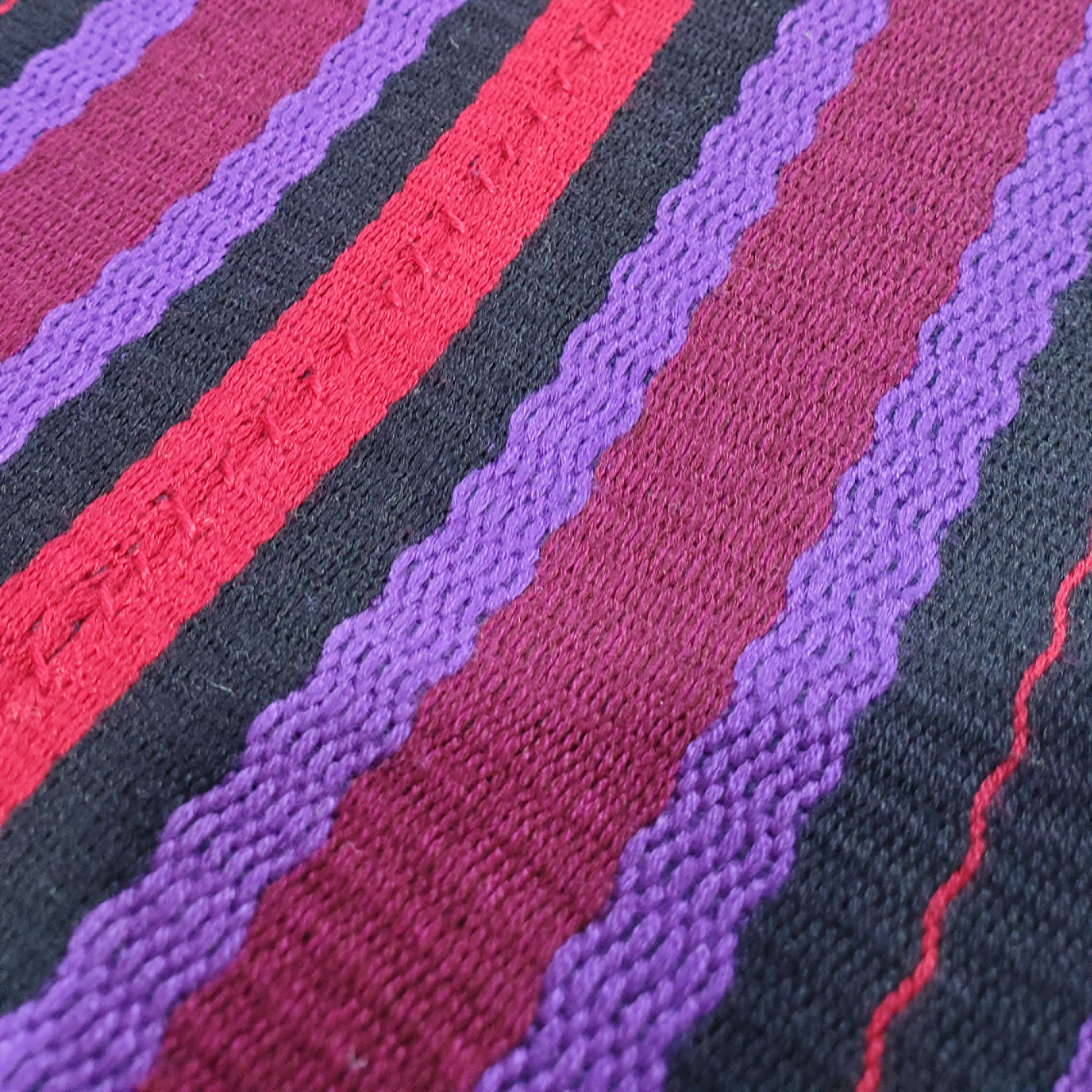

Supplement: S13 Fig — (TIF) [file pone.0325623.s014.tif]

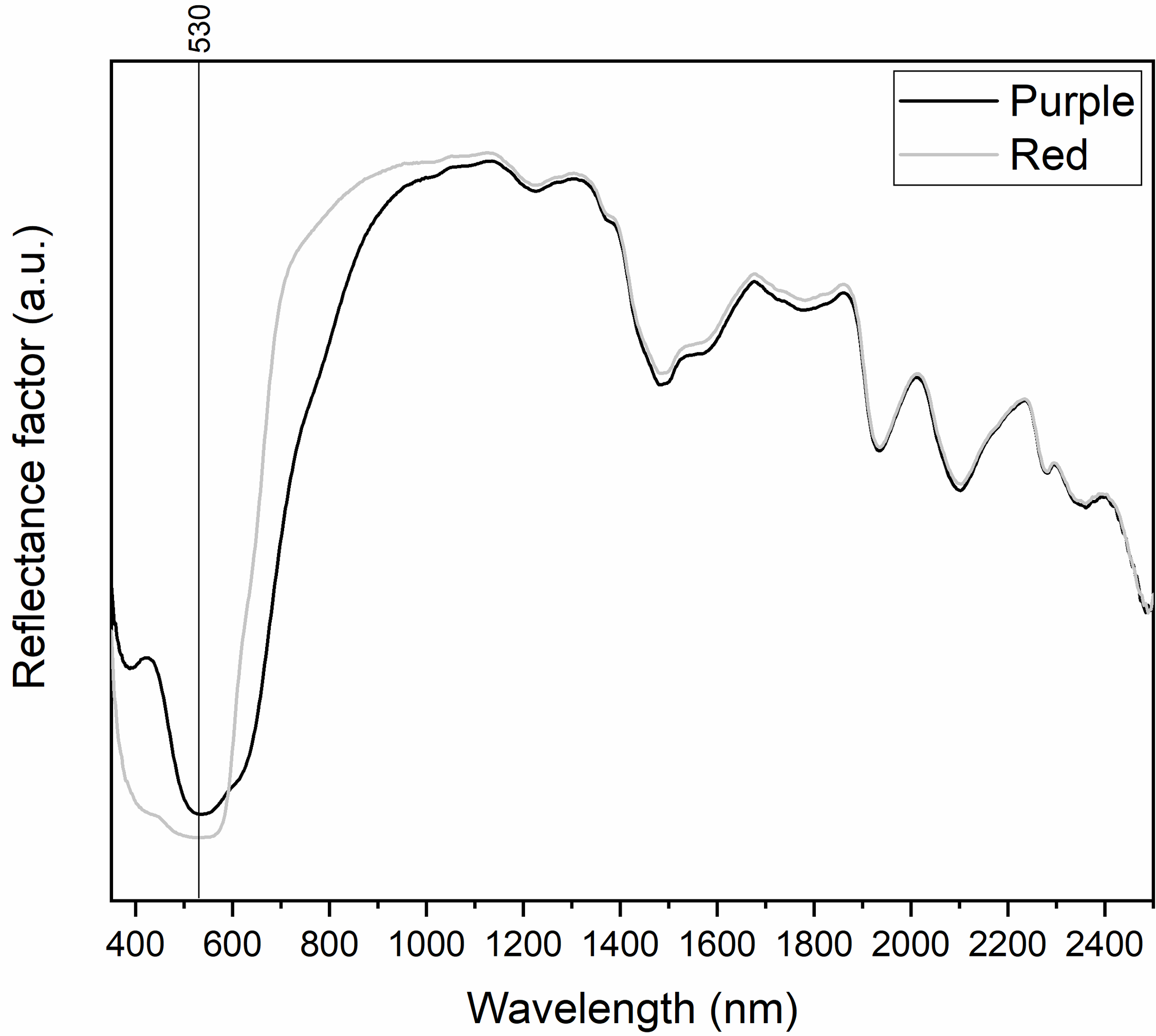

Supplement: S14 Fig — Purple areas were dyed with shellfish purple. (TIF) [file pone.0325623.s015.tif]

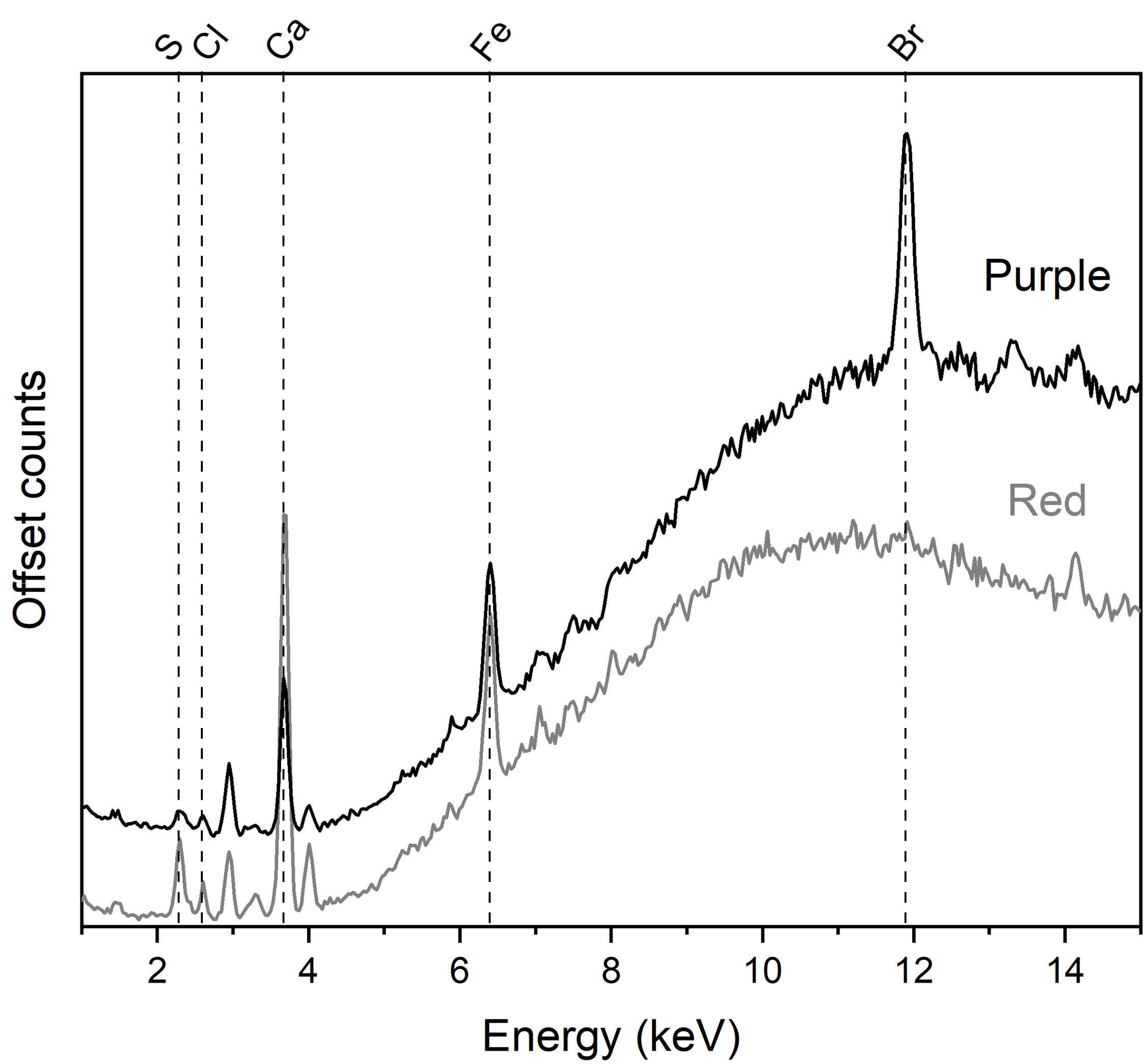

Supplement: S15 Fig — Purple areas were dyed with shellfish purple. (TIF) [file pone.0325623.s016.tif]
