# Supplementary material for: New insights into archaeological textiles (1000–1450AD) from the coastal region of the Atacama Desert: Preliminary evidence of a cochineal and shellfish purple dye combination
Source: PLoS One. 2025 Jun 4;20(6):e0325623. doi: 10.1371/journal.pone.0325623 (PMC12136422; doi:10.1371/journal.pone.0325623)
Supplement: S1 Table — Intensities correspond to K lines (Al - Mn). (DOCX) [file pone.0325623.s017.docx]

**Table S1**. XRF elemental peak intensities (counts) of the *in situ* soil analyses at PLM-3. Intensities correspond to K lines (Al - Mn).

| # | **Al** | **Si** | **S** | **Cl** | **K** | **Ca** | **Ti** | **V** | **Cr** | **Mn** |
| --- | --- | --- | --- | --- | --- | --- | --- | --- | --- | --- |
| **1** | 3004 | 69296 | 56350 | 307312 | 127148 | 2.61E+06 | 149452 | 7282 | 4698 | 54398 |
| **2** | 1689 | 59511 | 37388 | 266446 | 134098 | 2.91E+06 | 131706 | 6408 | 2808 | 43155 |
| **3** | 3040 | 59428 | 50971 | 360971 | 143683 | 2.38E+06 | 174746 | 7868 | 3105 | 51734 |
| **4** | 2580 | 54423 | 24446 | 308940 | 85989 | 3.70E+06 | 109743 | 5339 | 1439 | 36234 |
| **5** | 1800 | 64583 | 51055 | 206884 | 136970 | 2.22E+06 | 184810 | 8879 | 3950 | 58056 |
| **6** | 3665 | 58562 | 66187 | 566460 | 124684 | 2.08E+06 | 208567 | 8616 | 2802 | 58056 |
| **7** | 2684 | 68509 | 34542 | 215815 | 118354 | 3.08E+06 | 118095 | 5368 | 3281 | 49429 |
| **8** | 3655 | 70659 | 21680 | 237690 | 131442 | 2.67E+06 | 133343 | 5944 | 2126 | 39281 |
| **10** | 1479 | 57347 | 22732 | 276172 | 94756 | 4.54E+06 | 75860 | 723 | 2803 | 31158 |
| **11** | 2217 | 42095 | 28100 | 323621 | 80919 | 3.41E+06 | 110645 | 4400 | 2046 | 37892 |
| **12** | 2467 | 63905 | 19390 | 250572 | 118697 | 3.12E+06 | 117211 | 5241 | 2662 | 36777 |
| **13** | 1866 | 52138 | 63156 | 432300 | 90409 | 2.24E+06 | 219967 | 11164 | 3523 | 61833 |
| **14** | 2416 | 49217 | 46713 | 305387 | 92360 | 2.09E+06 | 260710 | 13654 | 6874 | 69494 |
| **16** | 2574 | 52629 | 69600 | 322354 | 113433 | 1.94E+06 | 286013 | 12890 | 3654 | 65749 |
| **17** | 3206 | 74115 | 53957 | 430147 | 111655 | 1.88E+06 | 120415 | 5183 | 3258 | 45119 |
| **18** | 2234 | 71074 | 52394 | 280047 | 126615 | 2.85E+06 | 162324 | 6221 | 3716 | 47484 |
| **19** | 2508 | 63152 | 59506 | 222766 | 131476 | 2.74E+06 | 200055 | 8530 | 4849 | 59687 |
| **21** | 2883 | 73854 | 36787 | 237212 | 150941 | 3.20E+06 | 117595 | 6012 | 2736 | 37564 |
| **22** | 2888 | 65001 | 51451 | 351688 | 166589 | 2.42E+06 | 179575 | 8127 | 2468 | 42000 |
