# Supplementary material for: New insights into archaeological textiles (1000–1450AD) from the coastal region of the Atacama Desert: Preliminary evidence of a cochineal and shellfish purple dye combination
Source: PLoS One. 2025 Jun 4;20(6):e0325623. doi: 10.1371/journal.pone.0325623 (PMC12136422; doi:10.1371/journal.pone.0325623)
Supplement: S2 Table — Intensities correspond to K lines (Fe - Zr). (DOCX) [file pone.0325623.s018.docx]

**Table S2**. XRF elemental peak intensities (counts) of the *in situ* soil analyses at PLM-3. Intensities correspond to K lines (Fe - Zr).

| # | **Fe** | **Ni** | **Cu** | **Zn** | **As** | **Rb** | **Sr** | **Y** | **Zr** |
| --- | --- | --- | --- | --- | --- | --- | --- | --- | --- |
| **1** | 3.34E+06 | 50022 | 41188 | 21878 | 7437 | 14703 | 297007 | 5007 | 71582 |
| **2** | 3.16E+06 | 48142 | 40206 | 20496 | 7150 | 15989 | 326385 | 4640 | 105142 |
| **3** | 3.72E+06 | 52104 | 41959 | 19632 | 7922 | 16121 | 279836 | 4678 | 58513 |
| **4** | 2.41E+06 | 48510 | 40344 | 16470 | 6490 | 12826 | 328279 | 7582 | 74328 |
| **5** | 4.08E+06 | 47096 | 37340 | 21559 | 8634 | 13913 | 259928 | 5778 | 81805 |
| **6** | 3.83E+06 | 52758 | 46233 | 21992 | 9097 | 12376 | 233555 | 5799 | 83639 |
| **7** | 2.93E+06 | 51476 | 41083 | 20258 | 7625 | 16143 | 327212 | 4287 | 54242 |
| **8** | 2.78E+06 | 50830 | 42897 | 22757 | 9651 | 19230 | 286533 | 4662 | 84246 |
| **10** | 1.81E+06 | 42805 | 40019 | 18496 | 6160 | 14222 | 370460 | 3215 | 46336 |
| **11** | 2.44E+06 | 46069 | 40281 | 19120 | 3361 | 12002 | 313134 | 3434 | 65510 |
| **12** | 2.65E+06 | 47827 | 43453 | 21647 | 5171 | 15555 | 297792 | 4147 | 80312 |
| **13** | 4.15E+06 | 48069 | 42007 | 19549 | 8107 | 9149 | 241387 | 5392 | 107156 |
| **14** | 5.14E+06 | 44141 | 36854 | 21084 | 7785 | 8195 | 228469 | 4560 | 86047 |
| **16** | 4.76E+06 | 45982 | 36538 | 19799 | 7620 | 9753 | 224879 | 4529 | 142117 |
| **17** | 2.97E+06 | 53733 | 47594 | 24846 | 10328 | 18161 | 270111 | 5205 | 85270 |
| **18** | 3.35E+06 | 51431 | 41766 | 20869 | 8345 | 13355 | 287507 | 5138 | 113480 |
| **19** | 3.81E+06 | 48719 | 38671 | 20615 | 8561 | 12192 | 269058 | 4534 | 97878 |
| **21** | 2.64E+06 | 49824 | 41932 | 18137 | 8850 | 18317 | 320173 | 4062 | 46992 |
